# Antiproliferative Homoleptic and Heteroleptic Phosphino Silver(I) Complexes: Effect of Ligand Combination on Their Biological Mechanism of Action

**DOI:** 10.3390/molecules25225484

**Published:** 2020-11-23

**Authors:** Khouloud Dammak, Marina Porchia, Michele De Franco, Mirella Zancato, Houcine Naïli, Valentina Gandin, Cristina Marzano

**Affiliations:** 1 Laboratoire Physico-Chimie de l’Etat Solide, Département de Chimie, Faculté des Sciences de Sfax, Université de Sfax, B.P. 1171, Sfax 3000, Tunisia; khoulouddammak04@gmail.com (K.D.); houcine_naili@yahoo.com (H.N.); 2 CNR-ICMATE, Corso Stati Uniti 4, 35127 Padova, Italy; 3 Department of Pharmaceutical and Pharmacological Sciences, University of Padova, via Marzolo 5, 35131 Padova, Italy; michele.defranco@phd.unipd.it (M.D.F.); mirella.zancato@unipd.it (M.Z.); cristina.marzano@unipd.it (C.M.)

**Keywords:** silver(I) complexes, phosphine ligands, cytotoxicity

## Abstract

A series of neutral mixed-ligand [HB(pz)_3_]Ag(PR_3_) silver(I) complexes (PR_3_ = tertiary phosphine, [HB(pz)_3_]^−^ = tris(pyrazolyl)borate anion), and the corresponding homoleptic [Ag(PR_3_)_4_]BF_4_ compounds have been synthesized and fully characterized. Silver compounds were screened for their antiproliferative activities against a wide panel of human cancer cells derived from solid tumors and endowed with different platinum drug sensitivity. Mixed-ligand complexes were generally more effective than the corresponding homoleptic derivatives, but the most active compounds were [HB(pz)_3_]Ag(PPh_3_) (**5**) and [Ag(PPh_3_)_4_]BF_4_ (**10**), both comprising the lipophilic PPh_3_ phosphine ligand. Detailed mechanistic studies revealed that both homoleptic and heteroleptic silver complexes strongly and selectively inhibit the selenoenzyme thioredoxin reductase both as isolated enzyme and in human ovarian cancer cells (half inhibition concentration values in the nanomolar range) causing the disruption of cellular thiol-redox homeostasis, and leading to apoptotic cell death. Moreover, for heteroleptic Ag(I) derivatives, an additional ability to damage nuclear DNA has been detected. These results confirm the importance of the type of silver ion coordinating ligands in affecting the biological behavior of the overall corresponding silver complexes, besides in terms of hydrophilic–lipophilic balance, also in terms of biological mechanism of action, such as interaction with DNA and/or thioredoxin reductase.

## 1. Introduction

In the last decades, in the search of metal-based antitumor drugs alternative to platinum-based chemotherapeutic agents, many metal complexes have been taken into consideration, and, among them, group 11 metal derivatives have shown encouraging perspectives. Whereas a plethora of different classes of copper(I,II) and gold(I,III) complexes have been investigated in the effort of circumventing platinum drug disadvantages [1,2], so far the antitumor activity of silver complexes has been less widely explored. On the other hand, anti-septic, anti-bacterial, and anti-inflammatory activities of silver compounds, such as silver salts, complexes and nanoparticles, are well known and extensively exploited in a large number of medical applications [3].

One of the greatest advantages in the clinical usage of silver compounds is their relatively low toxicity to humans that justify the pressing interest in the development of new silver-based drugs. More recently, silver complexes have also been shown to possess anticancer activity. Some excellent reviews summarize advances in this field [4,5,6].

Several classes of silver(I) derivatives display a strong in vitro antitumor activity against a variety of human cancer cell lines enhanced than that of the free ligands, indicating the importance of silver chelation, and often higher than the reference drug, cisplatin. The antiproliferative activity of silver complexes is strictly connected with their water stability, lipophilic balance, and rate of release of silver ions. These properties are ruled by the choice of suitable ligands that influences both the electronic and steric properties of the relative complexes [5,6].

A number of different ligands has been used to prepare silver complexes for anticancer purposes [3], but Ag(I) derivatives scoring the best results are those containing *N*-heterocycles carbenes, *N*-heterocycles, especially polypyridines, and P-donor tertiary phosphines or diphosphines [6]. Phosphine ligands are widely employed in transition metal coordination chemistry due to their versatility based on the nature of the substituents on phosphorus that regulates the physical and chemical properties of the metal complex. After the seminal paper published in 1988 by Berners-Price et al. [7], several phosphino silver derivatives endowed with significant antitumor activity, often associated to specificity toward carcinoma and sarcoma tumor cells as in the case of the cationic [Ag_2_{(S,S)-tetraphos}_2_](PF_6_) (tetraphos = bis{(2-diphenylphosphino-ethyl)phenyl phosphine}ethane) [8], have been described.

In the course of our studies on the cytotoxic activity of hydrophilic monocationic copper(I) compounds of the type [Cu(PR_3_)_4_]^+^ (PR_3_ = tris(hydroxymethyl)phosphine, thp; 1,3,5-triaza-7-phosphadamantane, PTA; tris(hydroxypropyl)phosphine, thpp), aiming at establishing structure–activity relationships, we have also investigated the efficacy of other coinage metal complexes, such as [Ag(PR_3_)_4_](PF_6_) and [Au(PR_3_)_4_](PF_6_) (PR_3_ = thp, PTA, thpp). [Ag(PR_3_)_4_](PF_6_) showed cytotoxic activities towards several human cancer cell lines slightly lower than related isostructural copper complexes, but, like copper and gold congeners, they were able to overcome cisplatin resistance [9]. 

Although the mechanism of action of silver-based anticancer agents has not been fully elucidated, it is now recognized that their anticancer action is based on different mechanisms compared to those of platinum derivatives, in terms of their DNA interaction and mitochondrial membrane targeting [5]. Berners-Price and coworkers observed a selective antimitochondrial activity for the tetrahedral diphosphino [Ag(eppe)_2_]NO_3_ derivative (eppe = diethyl-diphenyldiphosphinoethane) [10], and, more recently, various silver derivatives were found able to disrupt mitochondrial homeostasis, causing its imbalance and membrane depolarization [11,12]. This behavior, together with the reported ability of inactivating enzyme thiol groups with formation of mercaptides [13], could be related to an interaction between silver compounds and redox enzymes involved in maintaining healthy mitochondria homeostasis, such the selenoenzyme thioredoxin reductase (TrxR). TrxR is considered an attractive target for development of anticancer agents as it is frequently overexpressed in aggressive cancer cells [14]. On these basis, we previously investigated the ability of the homoleptic phosphino complex [Ag(PTA)_4_]PF_6_ to inhibit TrxR in vitro. It turned out that [Ag(PTA)_4_]PF_6_ was a very effective TrxR inhibitor, with half inhibition concentration (IC_50_)values in the nanomolar range suggesting that TrxR, recognized as the most relevant molecular target for gold species, could be an important target also for silver derivatives [9]. This behavior was also confirmed for other classes of cytotoxic silver derivatives, such as silver(I) *N*-heterocyclic carbene complexes [15,16,17]. 

In the recent past, following a systematic synthetic strategy to study the effect of charge, lipophilicity, hapticity and steric hindrance of the ligands on the biological behavior of Cu(I) complexes, we moved from cationic, homoleptic [Cu(PR_3_)_4_]^+^ complexes to neutral heteroleptic species containing *N*,*N*-bidentate chelate ligands and N,N,O- or N,N,N-scorpionate ligands [18,19]. Our investigations revealed that, among heteroleptic Cu(I) species, tris(pyrazolyl)borate derivatives [HB(pz)_3_]Cu(PR_3_) were the most promising compounds showing the strongest activity towards human cancer cell lines derived from solid tumors including relevant models of drug resistance (cisplatin and multidrug resistant cells). The obtained results clearly suggest that fine-tuning of the hydrophilic–lipophilic balance of the phosphine complexes may be important for maximizing their therapeutic potential.

**Scheme 1:**
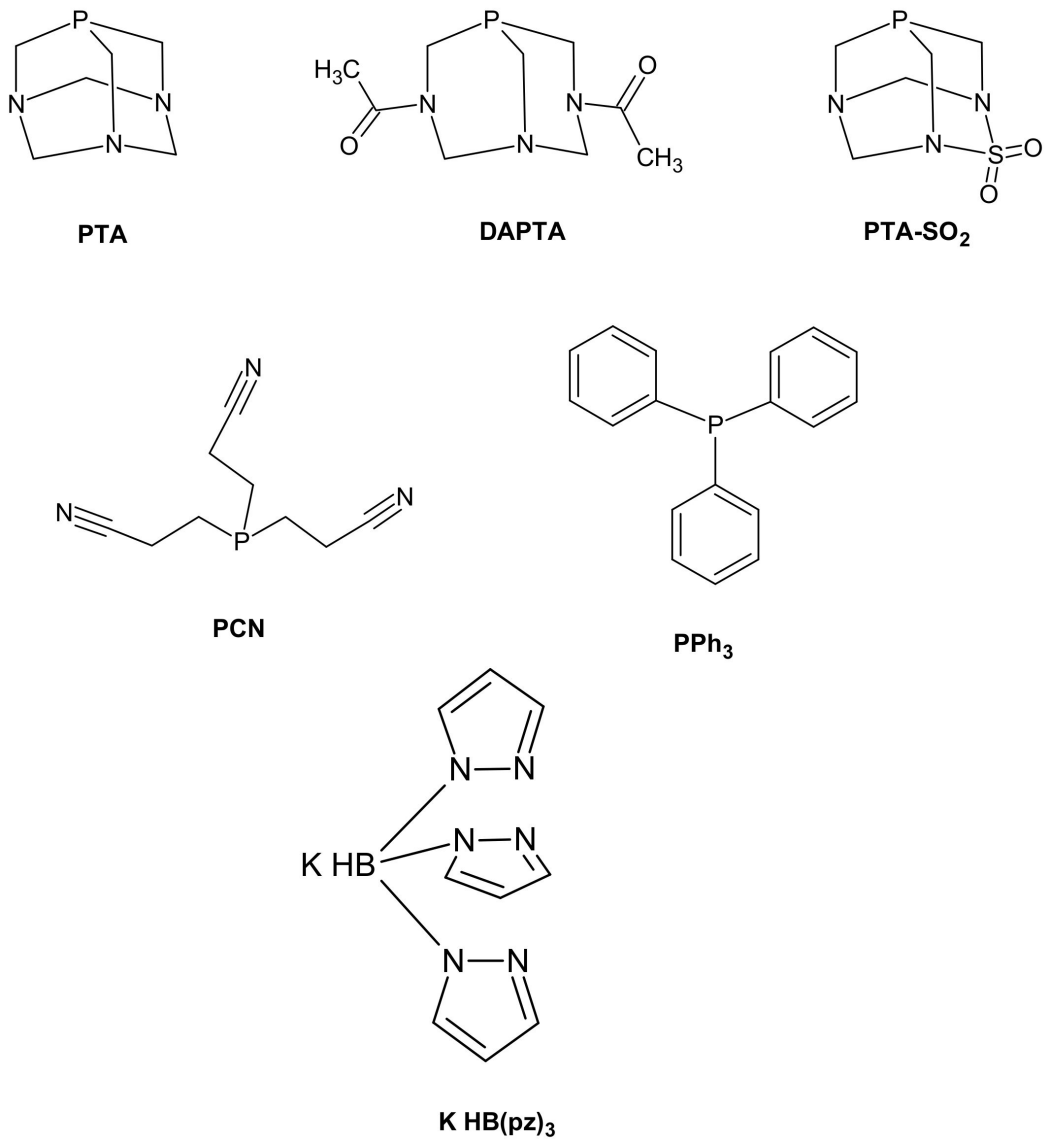
Structures of phosphine ^a^ and KHB(pz)_3_ ligands used in this work. ^a^ PTA = 1,3,5-triaza-7-phosphaadamantane; DAPTA = 3,7-diacetyl-1,3,7-triaza-5-phosphabicyclo [3.3.1]nonane; PTA-SO_2_ = 2-thia-1,3,5-triaza-phosphaadamantane 2,2-dioxide; PCN = tris(cyanoethyl)phosphine; PPh_3_ = triphenylphosphine.

**Figure 1:**
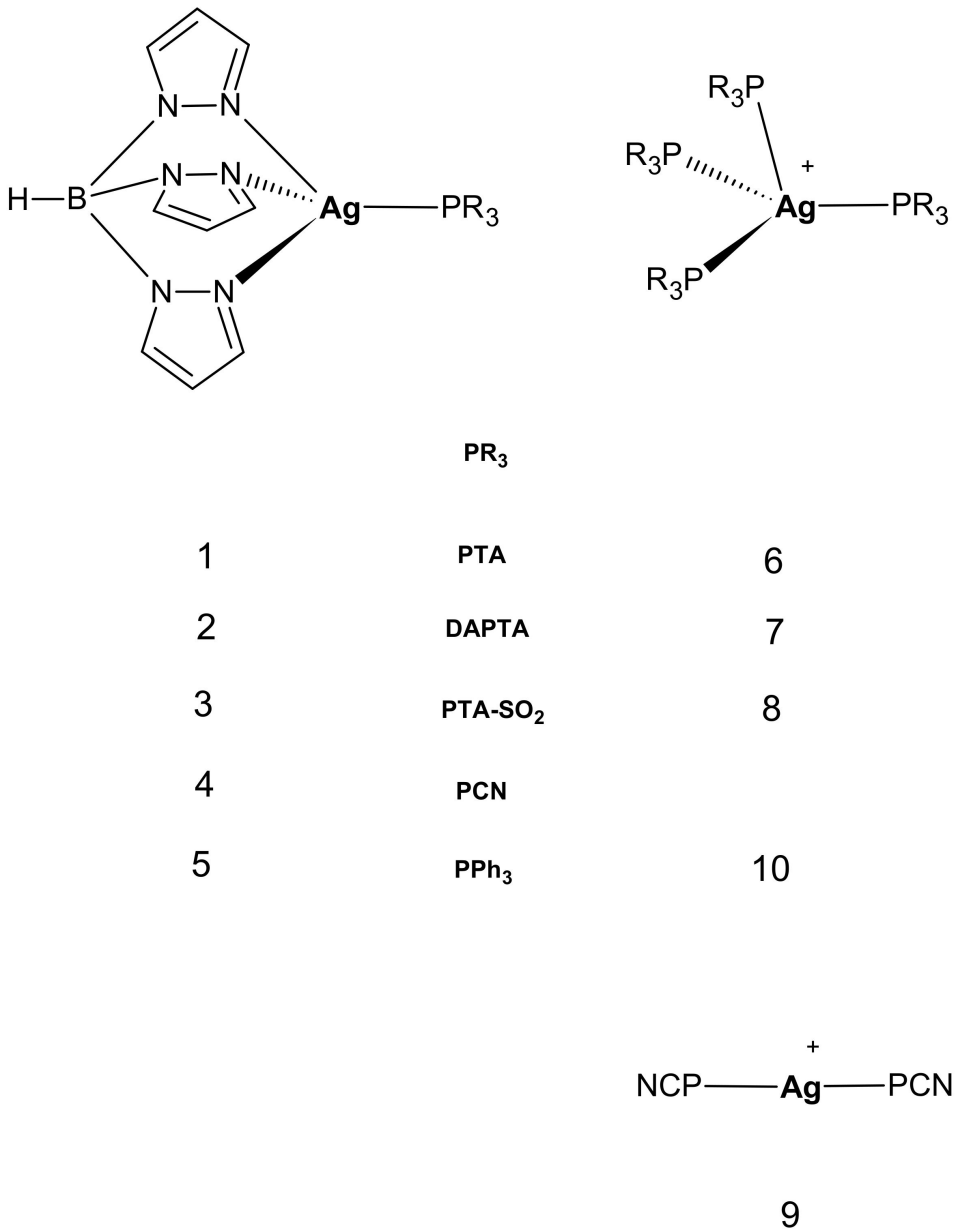
Structure of the heteroleptic and homoleptic complexes studied in this work.

Stimulated by the appealing results obtained with Cu(I) species, we now report on the synthesis and characterization of a series of analogous heteroleptic silver ‘3+1’ [HB(pz)_3_Ag(PR_3_)] complexes **1**–**5** (HB(pz)_3_ = tris(pyrazolyl)borate(1-); PR_3_ = 1,3,5-triaza-7-phosphaadamantane (PTA), complex **1**; 3,7-diacetyl-1,3,7-triaza-5-phosphabicyclo[3.3.1] nonane (DAPTA), complex **2**; PTA-SO_2_, 2-thia-1,3,5-triaza-phosphoadamantane 2,2 dioxide (PTA-SO_2_), complex **3**; tris(2-cyanoethyl)phosphine (PCN), complex **4**; triphenylphosphine (PPh_3_), complex **5**) (Scheme 1 and Figure 1). The antiproliferative properties of heteroleptic Ag(I) complexes were compared with those of related homoleptic, cationic [Ag(PR_3_)_4_]^+^ ones (Figure 1) with the aim to identify possible structure–activity relationships, as well as to get more information on the mechanism of action of Ag(I) phosphino complexes.

Recently, Pettinari and coworkers [20] reported on a pronounced anti-tumor activity (IC_50_ in the submicromolar range) against A375 human malignant melanoma cells of another class of heteroleptic, ‘3+1’-type [(tpms)Ag(PR_3_)] complexes comprising a tris(pyrazolyl)methanesulfonate tridentate chelate (tpms) in combination with mono-tertiary phosphines. The anticancer activity of these mixed-ligand derivatives was correlated to their ability to bind DNA.

Therefore, the main focus of our study was to explore the ability of the two series of Ag(I) complexes to interact with DNA and to hamper TrxR system directly on the purified proteins or on cell extracts. Biochemical assays monitoring cellular redox state and cellular morphological analysis by transmission electron microscopy (TEM) were performed to gain insight into the mechanism by which phosphino Ag(I) derivatives promote cancer cell death.

## 2. Results and Discussion

### 2.1. Synthesis and Characterization of Mixed-Ligand Silver Complexes

Silver(I) compounds **1**–**5** were prepared starting from the labile precursor [Ag(MeCN)_4_][BF_4_] by ligand exchange reactions in acetonitrile by addition of a stoichiometric amount of the relative phosphine and trispyrazolylborate salt. The complexes (white or light brown) are stable both in the solid and in the solution states, and moderately soluble in acetonitrile, dimethyl sulfoxide, and chloroform. Compounds **2** and **5** are slightly photosensitive and tend to become darker in the long run. Complexes **1**–**5** have been characterized by means of elemental analysis (C, H, N), multinuclear NMR (^1^H, ^31^P, ^13^C), IR spectroscopies, and high-resolution ESI(+) mass spectrometry. Unfortunately, no crystals suitable for X-ray characterization have been obtained except for compound **5** whose structure has been already published [21].

The infrared spectra of all compounds show the characteristic bands of the HB(pz)_3_(1-) chelate and phosphine ligands. In particular, derivatives **1**–**5** exhibit B-H stretching in the 2440–2500 cm^−1^ region, slightly shifted to higher frequency with respect to [K(HB(pz)_3_)] ligand (B-H 2434 cm^−1^) following the series PCN > PPh_3_ ≥ PTA-SO_2_> PTA = DAPTA with compound **4** showing the largest bathochromic shift (30 cm^−1^) (Figures S2 and S3). It is worth noting that also in the series of analogous [HB(pz)_3_]Cu(PR_3_) copper complexes, PCN derivative showed the largest shift of the B-H stretching frequency [19]. Other characteristic bands are due to C=O stretching at 1639 cm^−1^ (compound **2**); SO_2_ stretching at 1384 and 1187 (compound **3**); CN stretching vibrations of terminal cyano groups of PCN at 2254 cm^−1^ (compound **4**). Moreover, in the case of compounds **1**, **4** and **5** the band at ca. 1400 cm^−1^ can be assigned to the B-N stretching vibration.

^1^H NMR spectra of complexes **1**–**5**, recorded in DMSO or CDCl_3_ solution, show the signals relative to the pyrazolyl protons and to phosphine protons shifted with respect to the signals of the free ligands, generally with no loss of multiplicity or signal broadening (Figures S4 and S5). ^31^P NMR spectra are much more influenced by silver coordination and the signals are significantly downfield shifted when compared to those observed in uncoordinated phosphines. In particular, the shift variation between each free phosphine and the corresponding silver derivatives Δδ(^31^P) [Δδ(^31^P ) = δ (^31^P_complex_) − δ(^31^P_ligand_)] in CDCl_3_ goes from 22.8 ppm in the case of **5** to 35 ppm in the case of **4** (Table 1) suggesting a strong Ag-P interaction. Such values are greater with respect to those observed in isostructural [HB(pz)_3_]Cu(PR_3_) complexes (Δδ(^31^P) in the range 12–23 ppm) and for both metals, PCN phosphine determines the larger Δδ [19]. The shift can be correlated with the cone angle and the basicity of the phosphine [21] and is indicative of the strength of the metal–phosphorous bonding interaction. In the case of PCN, its peculiar umbrella conformation determines a wider cone angle with respect to those calculated for PPh_3_ and PTA (173° versus 145° and 103°, respectively [22,23]), and this feature can explain the significant variation of the ^31^P chemical shift.

At room temperature ^31^P NMR spectra of compounds **1**, **2** (in DMSO), **4** and **5** show a doublet or a doublet of doublets due to ^107/109^Ag-^31^P coupling (Figures S6, S8 and S9), differently from analogous [HB(pz)_3_]Ag(PR_3_) complexes containing trimesityl or tri (*o*-, *m*-, and *p*-) tolyl phosphine which display Ag-P coupling only at low temperature [21]. Both signal multiplicity and values of the Ag-P coupling constants are indicative of a strong Ag-P interaction and of stability of the complexes in solution. Detection of a broad signal in the ^31^P spectrum of **3** at room temperature could be due to equilibria of dissociation of PTA-SO_2_ ligand, but its low solubility prevented the possibility of performing NMR experiments at low temperature (Figure S7).

ESI(+)-MS spectra of all compounds were recorded in methanol (Figures S10 and S11). Full spectra of compounds **1**–**4** show signals corresponding to the [M + H]^+^ molecular ion peak with a very high relative abundance (50–100%). Sodiated [M + Na]^+^ ion peaks are detected in the case of compounds **1**, **2** and **4** (relative abundance 20–40%) whereas a small amount of the silver containing [M + Ag]^+^ species is formed with compounds **4** and **5** (relative abundance 5 and 10%, respectively). Peaks derived by loss of a pyrazolyl ring (*m*/*z* 68) from the molecular ion are present in the spectra of all compounds suggesting a common fragmentation pattern. The most abundant peak in the spectrum of **5** is relative to the adduct [M + Ag(PR_3_)]^+^ (*m*/*z* 953.1, [HB(pz_3_)]Ag(PPh_3_) + Ag(PPh_3_)]^+^). This rearrangement can be observed also in the case of **1** (*m*/*z* 742.9, [HB(pz_3_)]Ag(PTA) + Ag(PTA)], 30%), **2** (*m*/*z* 886.9, [HB(pz_3_)]Ag(DAPTA) + Ag(DAPTA)], 40%) and **3** (*m*/*z* 953.0, [HB(pz_3_)]Ag(PTASO_2_) + Ag(PTASO_2_)], 20%). The peak of phosphine-free cluster [(HB(pz)_3_Ag)_3_ + Ag]^+^ (*m*/*z* 1070.7) is the base peak in the spectra of **2** and **3**. Other clusters, such as [(HB(pz)_3_Ag)_2_ + Ag]^+^ (*m*/*z* 751.0) and [(HB(pz)_3_Ag)_2_ + H]^+^ (*m*/*z* 643.0), can also be evidenced at different extent in the spectra of compounds **1**–**4**. Peaks corresponding to the rearranged fragment [Ag(PR_3_)_2_]^+^ are detected in the spectra of **1**, **4** and, to a major extent, of **5** (*m*/*z* 631.2, [Ag(PPh_3_)_2_]^+^, 30%).

The presence of the molecular ion peak in the mass spectra of complexes **1**–**4** confirms what already suggested by NMR data: neutral heteroleptic silver complexes seem to retain their structure in solution both at millimolar and micromolar concentration without any evidence of ligand exchange and silver release, thereby remaining as intact neutral species that can be internalized in tumor cells by passive diffusion. Anyway, this feature should to be confirmed by more detailed solution studies.

### 2.2. Synthesis and Characterization of Homoleptic Phosphino Silver Complexes

Complexes **6**–**10** have been characterized by means of elemental analysis (C, H, N), multinuclear NMR (^1^H, ^31^P) spectroscopy, and high-resolution ESI(+) mass spectrometry. Cationic derivatives of **6**, **9** and **10** comprising a different counteranion have already been reported and characterized.

At room temperature, ^31^P NMR spectra of the newly synthesized tetrahedral complexes **7** and **8** show a singlet downfield shifted with respect to the free phosphine signal of 13.1 ppm (solvent D_2_O) and 7.4 ppm (solvent DMSO), respectively (Figures S12 and S13). Differently from heteroleptic complexes, no ^107/109^Ag-^31^P coupling was observed at room temperature probably due to fast exchange ligands. In ESI(+)MS spectra of both compounds no peaks related to [Ag(PR_3_)_4_]^+^ species can be detected, but only those related to [Ag(PR_3_)_2_]^+^ or [Ag(PR_3_)]^+^ species: [Ag(DAPTA)_2_]^+^ (*m*/*z* 565, 100%) and [Ag(PTA-SO_2_)(DMSO)]^+^ (*m*/*z* 392, 100%) (Figures S14 and S15). It is well known that phosphino silver derivatives easily undergo both fast ligand exchange in solution [24,25], so that NMR may also not detect mixtures of Ag(I) complexes, and speciation processes in diluted solution. We recently reported in depth studies concerning the solution chemistry of [Ag(PTA)_4_]^+^,[26,27] and [Cu(PR_3_)_4_]^+^ [28] derivatives in diluted systems, as those utilized for biological studies. A partial dissociation of [Ag/Cu(PR_3_)_4_]^+^ species was observed leading to the formation of [Ag/Cu(PR_3_)_n_]^+^ derivatives (n = 1–3). In particular, in the case of [Ag(PTA)_4_]^+^ the predominant species in solution at micromolar concentration, typical of the in vitro biological assays, was found to be the linear [Ag(PTA)_2_]^+^. The dissociation into lower stoichiometry species has been directly correlated to the triggering of the antitumor activity of copper compounds [29], according to the hypothesis that coordinative unsaturated species could be the active species [30]. A similar behavior can be hypothesized also for [AgP_4_]^+^ complexes.

### 2.3. Cytotoxicity against Cultured Human Cancer Cells

Homoleptic and heteroleptic silver(I) complexes as well as uncoordinated tris(pyrazolyl) borate and phosphine ligands were tested for their cytotoxic activity by means of the MTT assay, as reported in the Experimental Section. The in-house human cancer cell line panel contains examples of pancreatic (BxPC3), colon (HCT-15), breast (MCF-7), cervical (A431), and lung (A549) cancers as well as of melanoma (A375). Cisplatin was used as reference compound and was tested under the same experimental conditions. The cytotoxicity parameters, in terms of IC_50_ obtained after 72 h of exposure, are listed in Table 2.

Free ligands showed negligible cytotoxicity, with mean IC_50_ values ranging from about 30 to over 100 µM. On the contrary, all tested silver(I) compounds showed a significant in vitro antitumor potential, with IC_50_ values in the low-micromolar range.

As a general consideration, neutral heteroleptic complexes **1**–**5** were, on average, more effective than the corresponding homoleptic cationic complexes **6**–**10**. As an example, over the six employed cancer cell lines, [HB(pz)_3_]Ag(PTA-SO_2_) **3** elicited average IC_50_ value of 5.9 µM compared with average IC_50_ of 9.8 µM obtained with its homoleptic counterpart [Ag(PTA-SO_2_)_4_][BF_4_] **8**. In both complex series, the cytotoxicity profile generally follows the same activity trend PTA~DAPTA < PTA-SO_2_ < PCN < PPh_3_, i.e., **1**~**2** < **3** < **4** < **5** and **6**~**7** < **8** < **9** < **10**.

Complexes **1**–**5** were more cytotoxic than the reference compound cisplatin against human pancreatic (BxPC3), colon (HCT-15) and breast (MCF-7) cancers. Among all, derivative **5** emerged as the most cytotoxic one with mean IC_50_ values against all tested cancer cell lines about 3 times lower compared to those calculated for cisplatin (average IC_50_ values of 2.5 and 7.9 µM, respectively). Against HCT-15 cells, **5** was about 7 times more effective than cisplatin.

On the other hand, homoleptic cationic complexes **6**, **7** and **8** possessed a lower antiproliferative effect compared with that of the reference chemotherapeutic drug, whereas compounds **9** and **10** were more effective than cisplatin towards almost all tested cancer cells. In particular, against human colon HCT-15 and pancreatic BxPC3 cancer cells complexes **9** and **10** were roughly 4.4- and 3.4-fold more effective than cisplatin, respectively.

The in vitro antitumor effect was also assessed on a human ovarian cancer cell line pair which has been selected for sensitivity/resistance to cisplatin (2008/C13* cancer cells). The cisplatin responsive 2008 cells were derived from a patient with cystadenocarcinoma of the ovary and the cisplatin-resistant counterpart, C13* cells, was generated by monthly selection with low doses of cisplatin [31]. The most important molecular mechanisms involved in drug resistance of C13* cancer cells comprise high cellular glutathione and TrxR levels, reduced cellular drug uptake and/or an enhanced DNA damage repair. Cell killing effects in sensitive and resistant cells were evaluated after 72 h of treatment by MTT test. The cross-resistance profiles were evaluated by means of the resistance factor (R.F.), which is defined as the ratio between IC_50_ values for the resistant cells and those arising from the sensitive ones (Table 3).

All tested silver(I) complexes proved to be equally effective against cisplatin-sensitive and -resistant cell lines, with RFs about 10 times lower than that of cisplatin. This result attested the ability of these species to overcome the acquired cisplatin resistance, ruling out the occurrence of cross-resistance phenomena.

### 2.4. Mechanistic Studies

As many studies identified DNA as a putative molecular target for silver(I) complexes [20], we firstly evaluated the ability of Ag(I) complexes to interact with isolated CT-DNA by UV absorption and fluorescence titrations.

Upon increasing concentration of heteroleptic compound **5**, a dose-dependent modification of the DNA absorption bands was detected (see Figure S16A). In particular, a slight hyperchromic effect at 263 nm was evidenced, indicative of an electrostatic interaction or partial destabilization of DNA chain. Conversely, no significant modifications in DNA properties were detected after incubation with homoleptic cationic silver(I) complex **10**, thus, suggesting lack of DNA interactions. In order to further confirm the binding of compounds **1**–**5** to DNA, binding affinities of silver(I) complexes have been monitored via competitive binding studies using ethidium bromide displacement assay. Increasing amounts of test compounds were added to the DNA-EB complex and the emission spectra were recorded (data not shown). From the observed spectra, K_SV_ was calculated according to the classical Stern–Volmer equation (Figure S16B). The slope of the regression curves clearly indicated the binding activity on DNA for **1**–**5**, whereas a scarce ability of interacting with DNA was confirmed for **6**–**10**.

**Figure 2:**
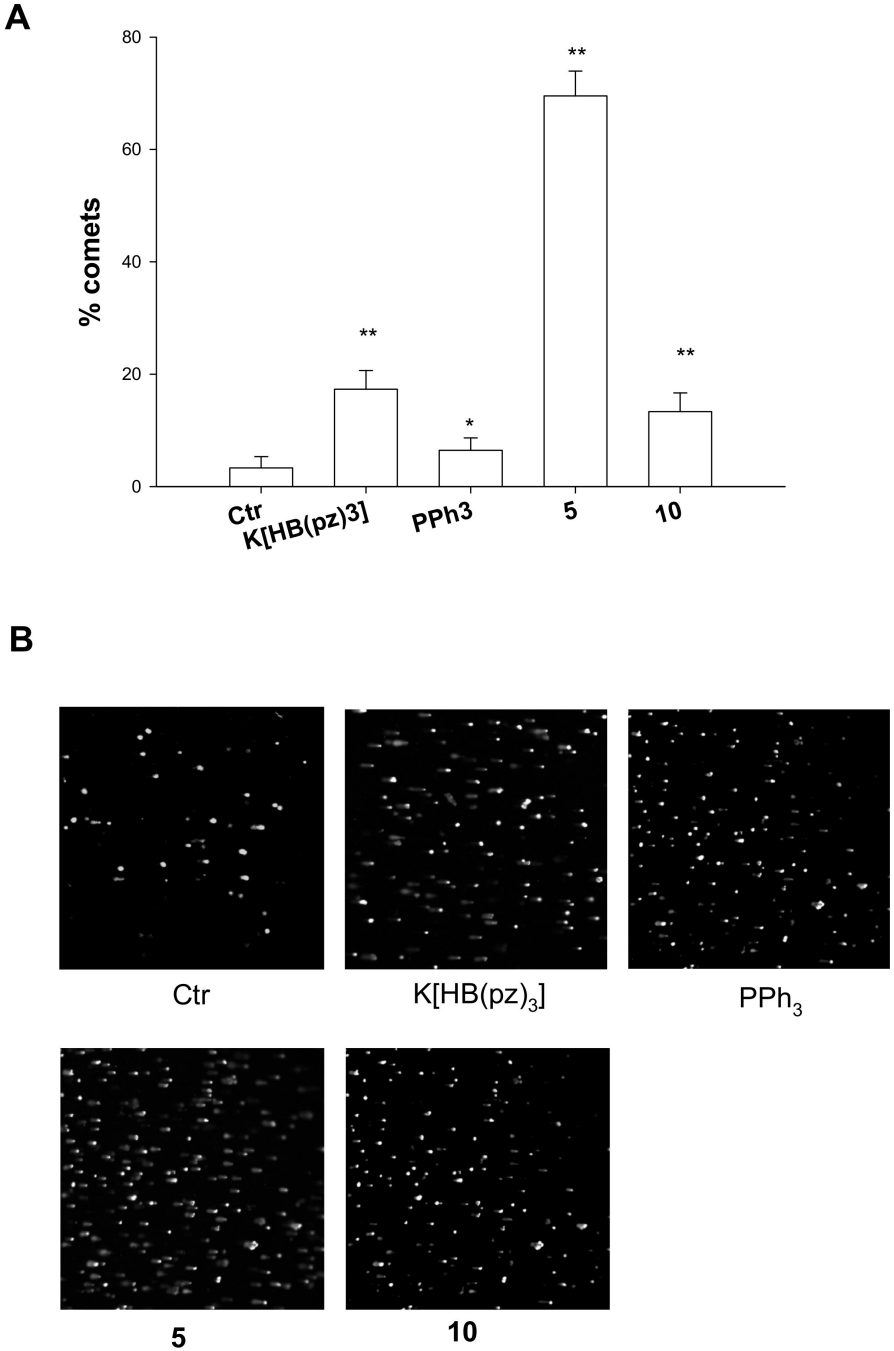
Comet assay; 2008 cells were treated for 6 h with IC_50_ values of tested compounds and then processed for comet assay. (**A**) Number of cells with well-formed comets measured by ImageJ software. The error bars indicate the S.D. * *p* < 0.01; ** *p* < 0.05(**B**) Representative images of 2008 cells.

Coherently, cell studies assessing DNA damage by using alkaline single cell gel electrophoresis (Comet assay) revealed a marked ability in causing cellular DNA fragmentation as expression of a direct DNA damage for the heteroleptic [HB(pz)_3_]Ag(PPh_3_) **5**. As can be seen from Figure 2 (panels A and B), showing the results obtained with human ovarian 2008 cancer cells treated with IC_50_ doses of **5** or **10** for 6 h, **5** displayed an increase of about 69% of well-formed comets, whereas **10** was barely effective in provoking DNA damage. It is interesting to note than uncoordinated HB(pz)_3_ induced itself a substantial increase in electrophoretic migration of the DNA fragments. On the contrary, uncoordinated phosphine PPh_3_ was scarcely effective in increasing comet tail lengths.

In recent years, several different metal-based compounds have been found to exert their biological activities via DNA-independent mechanisms, involving enzyme inhibition pathways, and recent studies pointed out that TrxR is a potential biomolecular target for many metal-based drugs.

**Figure 3:**
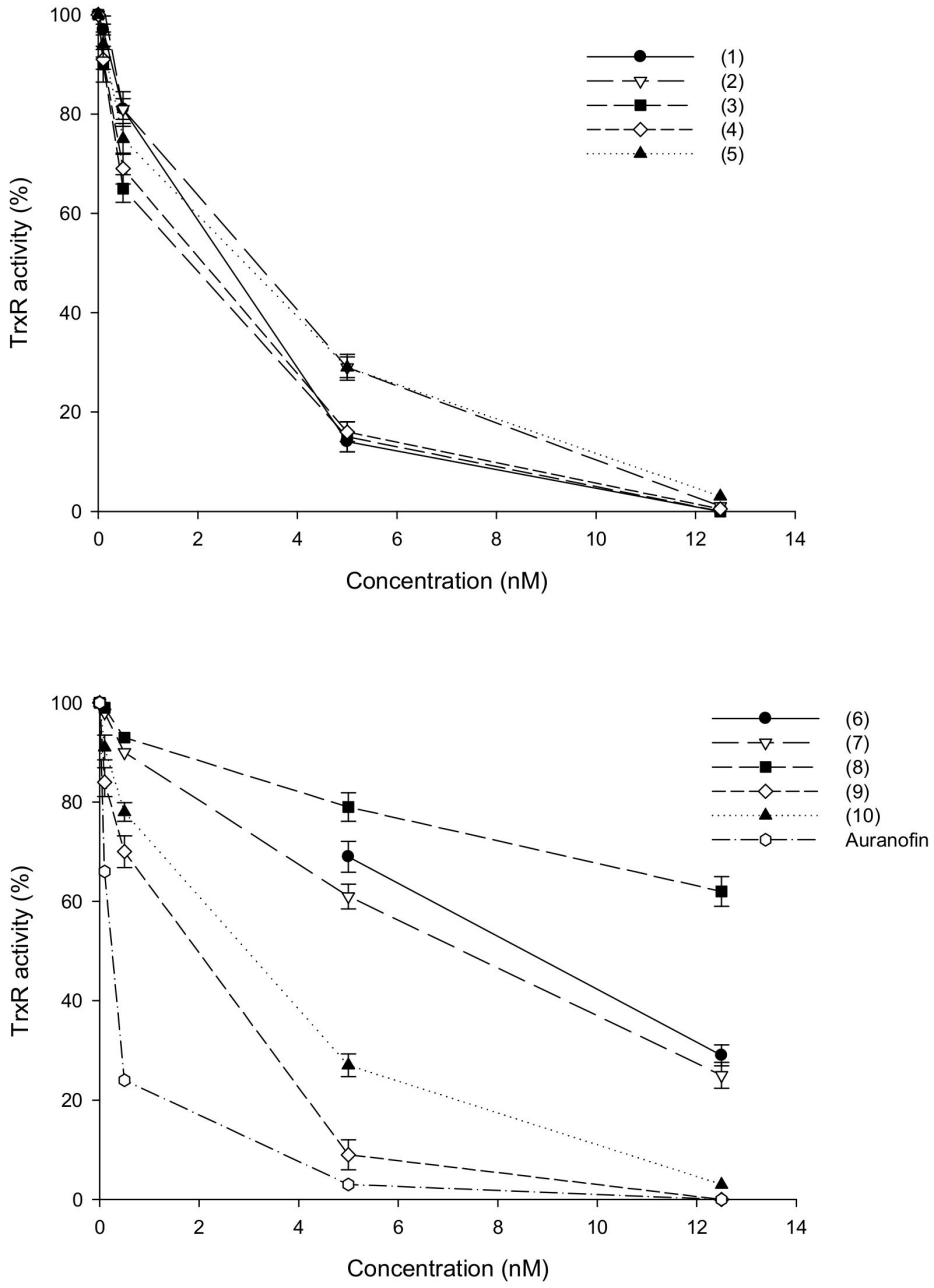
Effects of silver(I) complexes on human TrxR1. Thioredoxin reductase 1 activity was assayed by measuring NADPH-dependent reduction of DTNB at 412 nm as described in the Experimental Section. Error bars indicate S.D.

Au(I) and Ag(I) complexes were described as effective inhibitors of redox-active Sec-containing TrxR [32,33,34]. Based on these findings, we tested homoleptic and heteroleptic silver(I) complexes as potential TrxR inhibitors. Compounds **1**–**10** were initially evaluated for their inhibitory potential toward human TrxR1 in cell-free systems, according to established protocols as described in the Experimental Section. Ag(I) complexes were tested at increasing concentrations (0.1–10 nM range) and their activity was compared to that of auranofin, a phosphine Au(I) thiolate complex acting as potent TrxR enzyme inhibitor. Dose−effect response curves are reported in Figure 3 and the IC_50_ values calculated from the dose-effect curves by means of a four parameter logistic model are listed in Table 4.

All uncoordinated ligands tested in the 5–100 nM range were completely ineffective in hampering TrxR1 activity (data not shown). Conversely, the isolated human cytosolic TrxR1 appeared markedly inhibited by silver(I) complexes (Figure 3) and IC_50_ values for all tested derivatives were in the low-nanomolar level (2.4–16.8 nM range). Among all, hydrophilic homoleptic compounds **6**–**8** were the less effective derivatives, showing half-maximal inhibitory concentrations from 2.5 to 5.5 times higher than those of the other tested Ag(I) complexes (Table 4). Moreover, Ag(I)NO_3_ was effective in inhibiting the redox enzyme activity at nanomolar level, thus confirming the hypothesis that Ag(I) can target the selenocysteine residue of TrxR [32].

**Figure 4:**
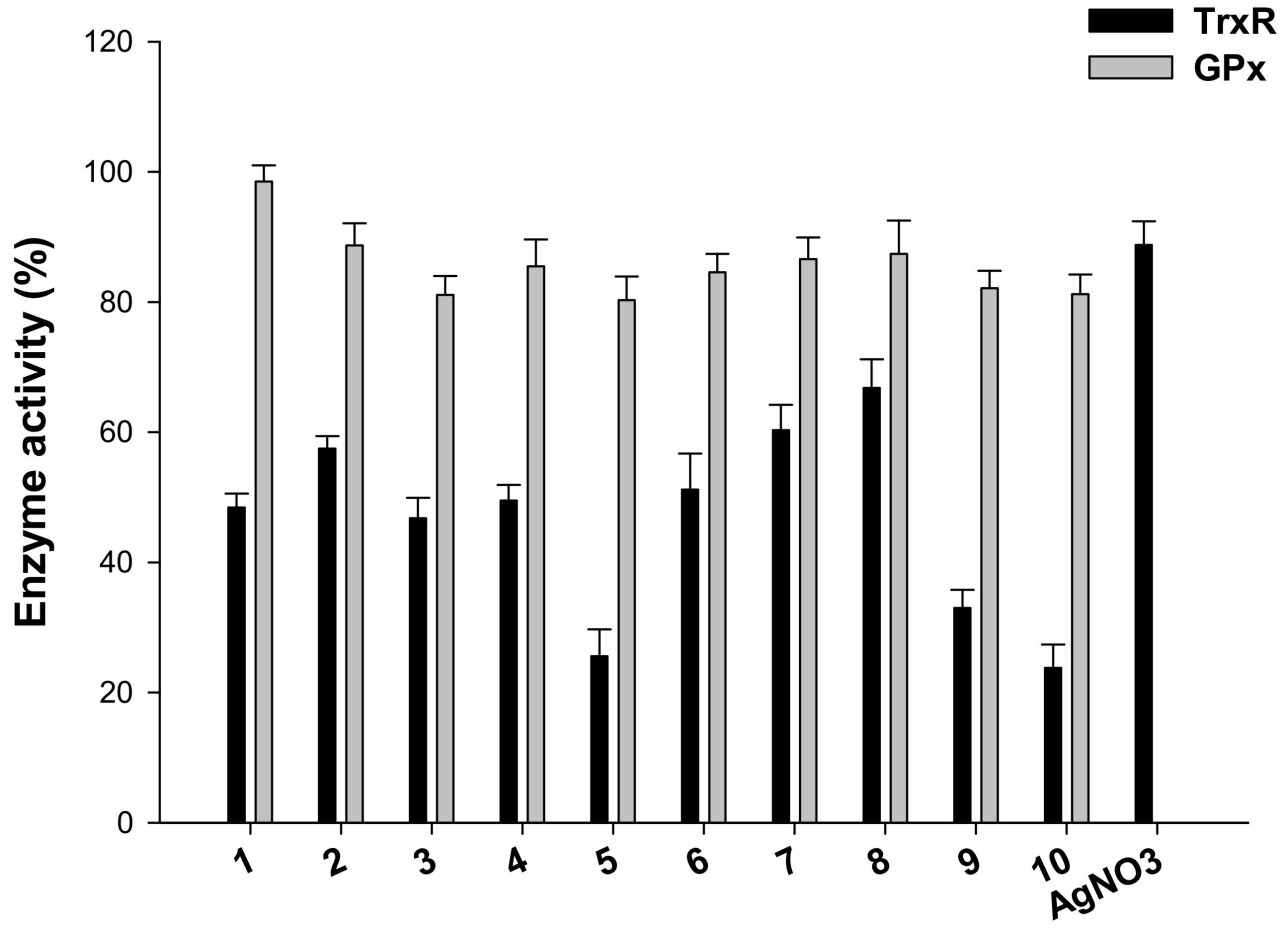
Effects of compounds **1**–**10** and AgNO_3_ on redox enzymes in human ovarian cancer cells. The 2008 cells were incubated for 18 h with tested compounds. Subsequently, cells were washed twice with Phosphate Buffer Solution (PBS) and lysed. TrxR activity was tested by measuring NADPH-dependent reduction of DTNB and glutathione peroxidase (GPx) activity was followed at 340 nm.

TrxR inhibition was also evaluated in 2008 cells treated for 18 h with equimolar concentrations (2.5 µM) of all phosphino silver(I) derivatives and of AgNO_3_. TrxR activity was assayed by measuring at 412 nm NADPH-dependent reduction of DTNB (Figure 4).

It is worth noting that all derivatives were able to decrease cellular TrxR activity of about 50%. Among all, compounds **5** and **10** were the most effective in hampering selenoenzyme catalytic activity, inducing about 75% reduction of total cellular TrxR activity. In addition, homoleptic cationic Ag(I) complex **9** was able to reduce cellular redox TrxR activity by about 70%. Conversely, AgNO_3_ tested under the same experimental condition was less effective with respect to all tested derivatives, being able to decrease TrxR activity only by 12%.

Silver(I) compounds were also evaluated as potential inhibitors of glutathione peroxidase GPx (GPx), a TrxR closely related Sec-containing redox enzyme. Interestingly, none of the tested complexes was effective in significantly decreasing GPx activity, thus attesting a preferential activity towards TrxR enzyme (Figure 4). Overall, these results on redox enzymes are in line with previously reported studies on other Ag(I) complexes and point out the selenoenzyme TrxR as a leading molecular target for silver(I) complexes.

The TrxR/Trx system plays a central role in the multiple cellular redox regulation networks and acts as an important modulator of tumor development [33]. Inhibition of this redox regulatory system has been shown to determine loss of cellular redox homeostasis either in terms of sulfhydryl redox status and increase in cellular basal production of Reactive Oxygen Species (ROS), thus, inducing cancer cell death [34].

**Figure 5:**
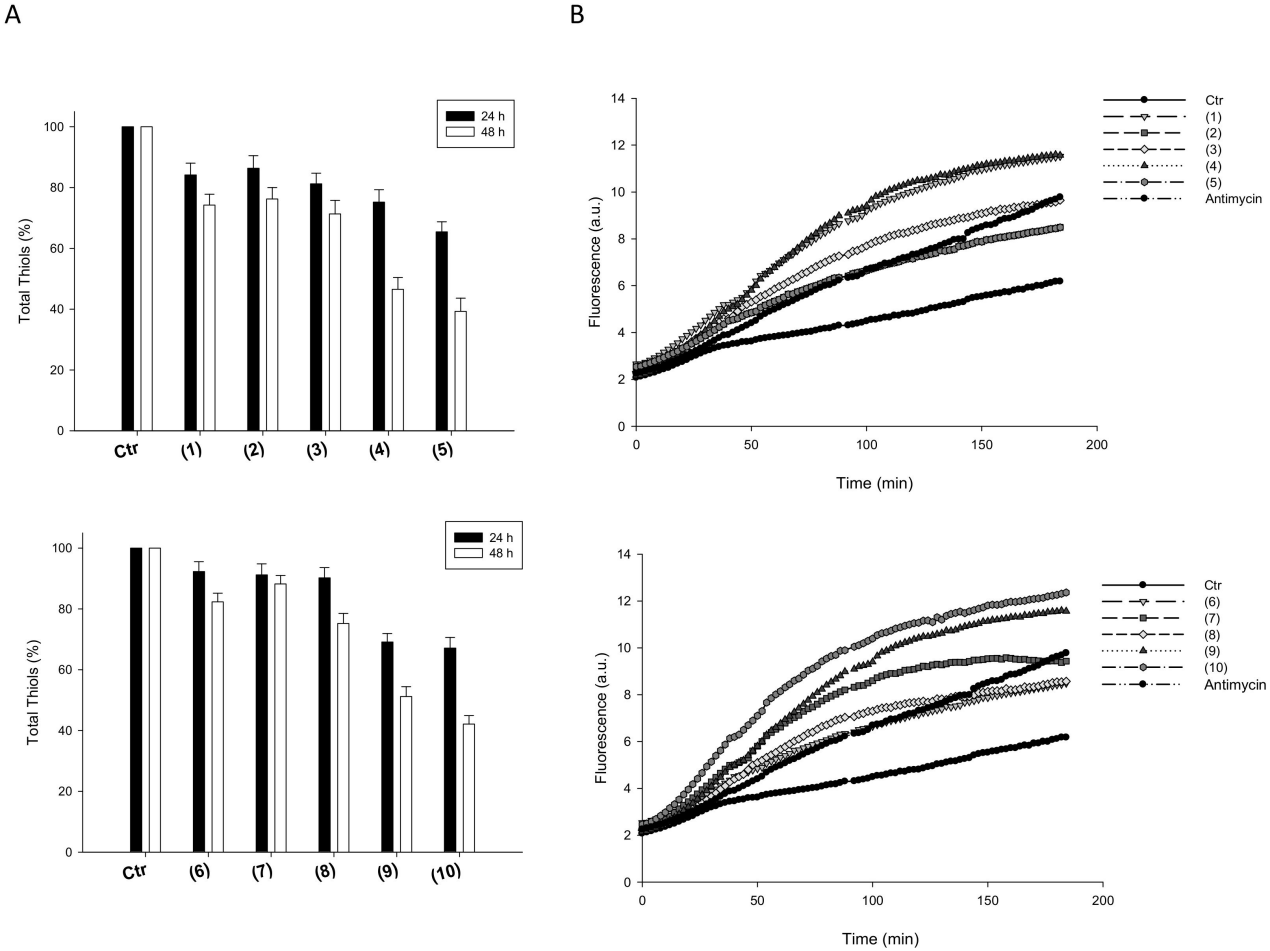
Cellular total thiol state and ROS production. (**A**) Sulfhydryl content in 2008-treated cancer cells incubated for 24 h or 48 h with tested silver(I) compounds. The sulfhydryl group amount was determined by the DTNB assay. Error bars indicate S.D. (**B**) effect of silver(I) compounds on hydrogen peroxide formation in 2008 cells. 2008 cells were pre-incubated in PBS/10 mM glucose medium for 20 min at 37 °C in presence of 10 µM CM-DCFDA and then treated with IC_50_ of silver(I) compounds.

On these bases, the effect induced by silver(I) complexes on total cellular sulfhydryl content and basal cellular ROS production were assayed in 2008 human ovarian cancer cells (Figure 5).

Intracellular thiols were significantly decreased in a time-dependent manner after treatment with all tested silver(I) complexes. Interestingly, 2008 cancer cells treated with compounds **5** and **10** for 48 h showed the lowest levels of cellular sulfhydryl groups compared to control cells (Figure 5, panel A).

Coherently, treatment of 2008 cells, with either homoleptic or heteroleptic silver(I) complexes, determined a substantial time-dependent increase in cellular basal ROS production (Figure 5, panel B). Notably, treatment with **5** and **10** determined an increase in basal hydrogen peroxide formation rather similar to that obtained with antimycin, a classic inhibitor of the mitochondrial respiratory chain at the level of complex III.

These latter results indicating that the newly developed silver(I) induce an oxidative shift in the redox status of 2008 cells are consistent with their ability to target TrxR.

Induction of oxidative stress and increase ROS production can in turn prompt the collapse of mitochondrial membrane potential as well as loss of mitochondrial shape and integrity (*swelling*). We hence evaluated the effect determined by treatment with silver(I) complexes in terms of modification of mitochondrial pathophysiological characteristics, such as mitochondrial membrane potential and morphological changes. In particular, 2008 cells were treated for 24 or 48 h with IC_50_ concentrations of tested Ag(I) complexes, and the percentage of cells with hypopolarized mitochondrial membrane potential was determined fluorimetrically by means of the Mito-ID^®^ Membrane Potential Kit. In addition, TEM analyses were performed on 2008 cells after 48 h treatment with IC_50_ concentrations of the most effective silver(I) complexes **5** and **10**.

**Figure 6:**
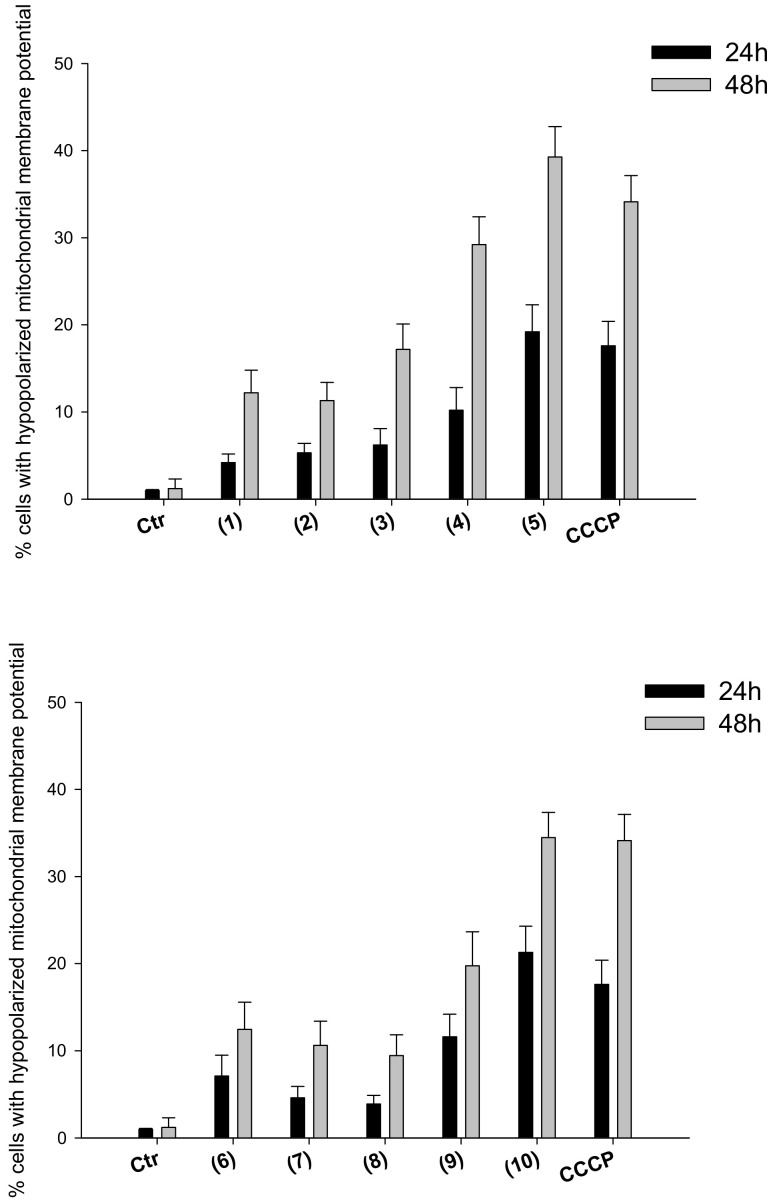
Mitochondrial membrane potential. Effects of silver(I) complexes on mitochondrial membrane potential. 2008 cells were treated for 24 h or 48 h with IC_50_ concentrations of tested complexes or CCCP (3 μM). The percentage of cells with hypopolarized mitochondrial membrane potential was determined by Mito-ID^®^ Membrane Potential Kit. Data are the means of three independent experiments. Error bars indicate S.D.

As summarized in Figure 6, a significant time-dependent increase of cells with depolarized mitochondria was observed after treatment with tested complexes. Again, compounds **5** and **10** were confirmed as the most effective ones in the two series, being able to induce an increase in the percentage of hypopolarized cells similar to that induced by reference compound carbonyl cyanide-*m*-chlorophenylhydrazone (CCCP).

**Figure 7:**
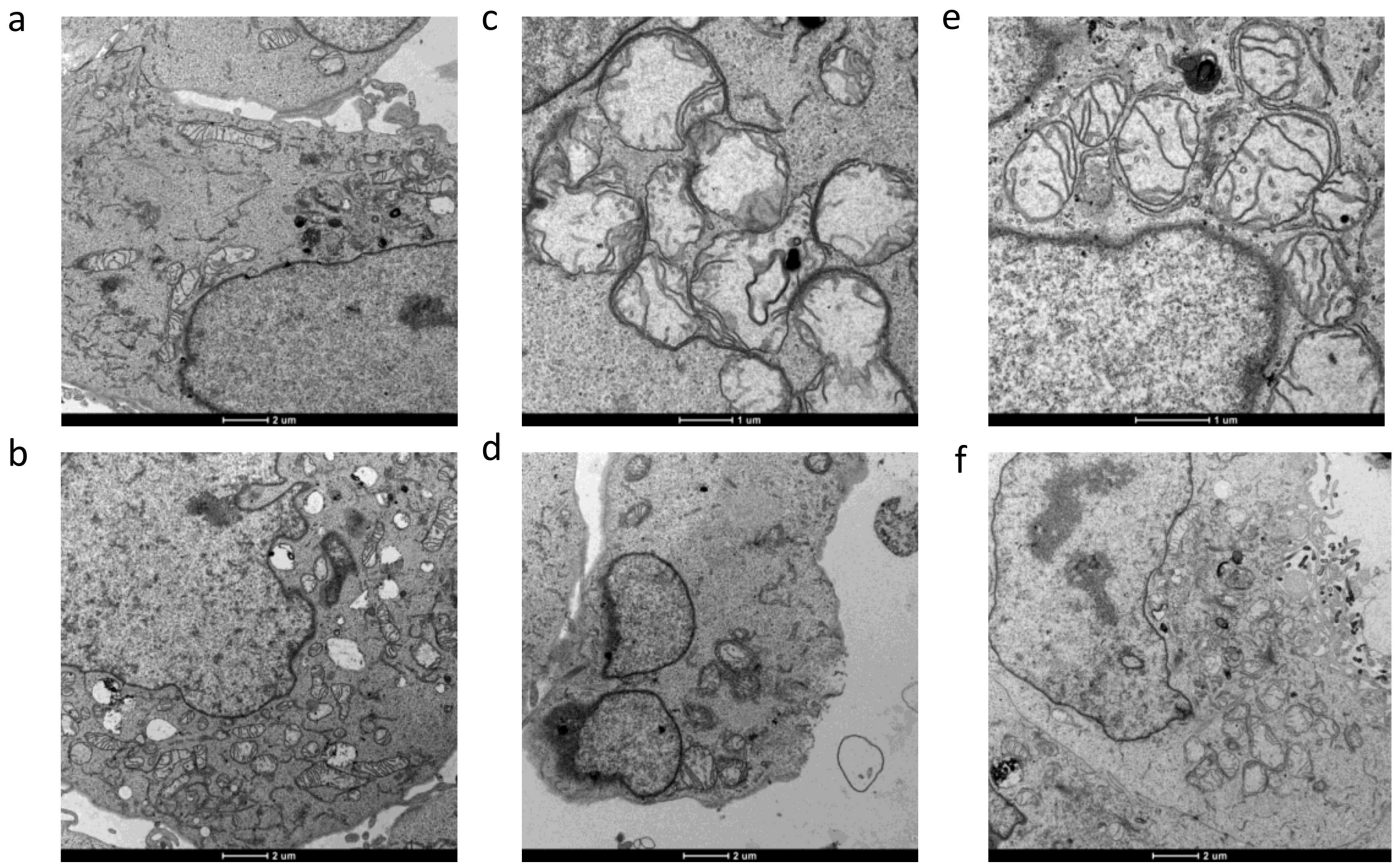
TEM analysis. Transmission electron micrographs of 2008 cells after 48 h treatment with silver(I) complexes (**5**) and (**10**). (**a**) and (**b**): controls; (**c**,**d**): (**5**); (**e**) and (**f**): (**10**).

Accordingly, morphological analyses obtained by TEM of 2008 cells treated with both silver(I) derivatives **5** and **10** showed mitochondria with disrupted cristae and a significant increase in volume (swelling) with respect to control cells, confirming that **5** and **10** elicited a substantial modification of mitochondria physiology (Figure 7).

In addition, cells treated with silver(I) derivatives exhibited the characteristic ultrastructural features of apoptosis, such as cell shrinkage, and chromatin condensation.

**Figure 8:**
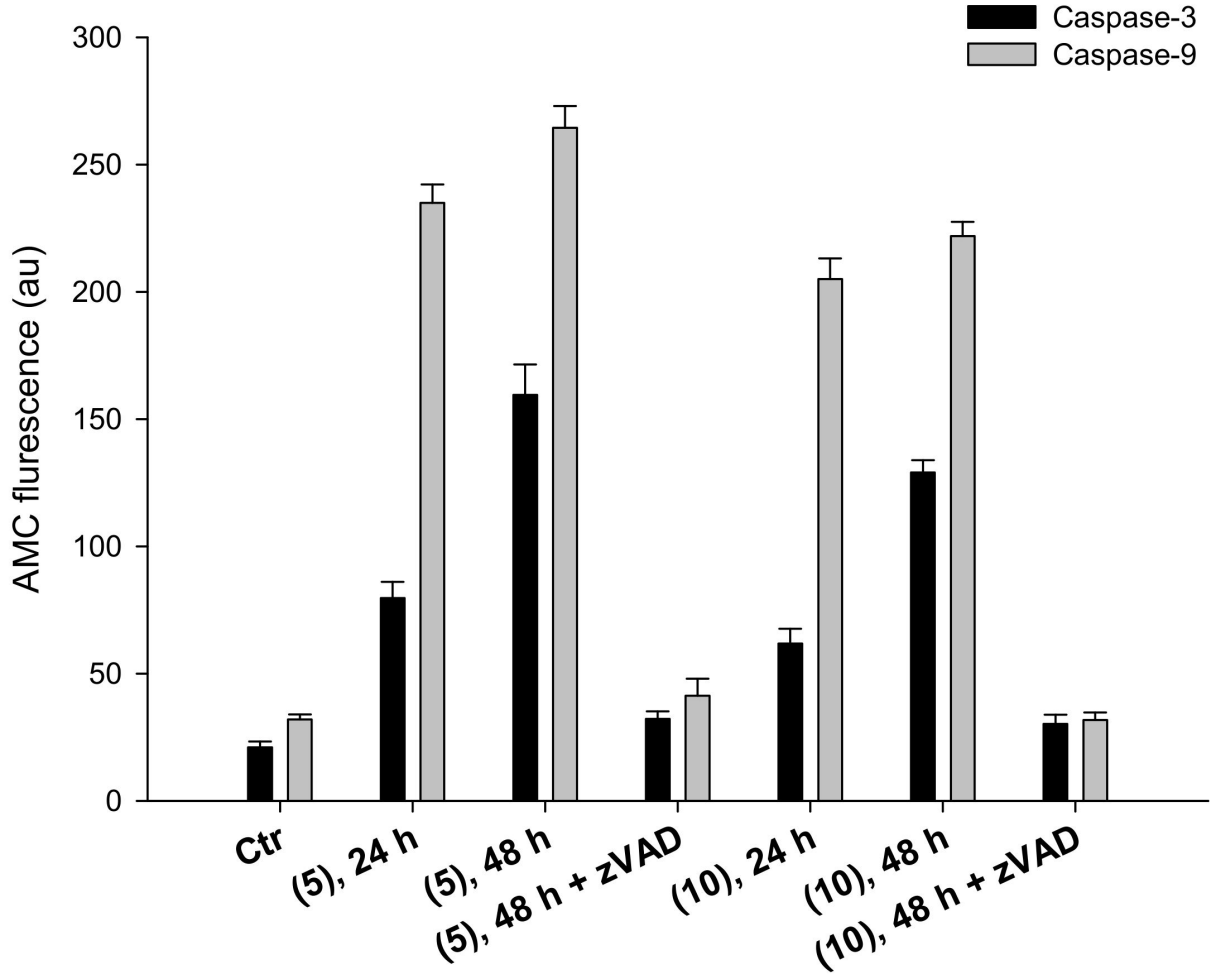
Caspase-3 and -9 activation. Effect of silver(I) compounds on caspase-3 and -9 activation. 2008 cells were incubated for 24 or 48 h with IC_50_ of compounds (**5**) or (**10**), broad-spectrum caspase inhibitor zVAD, and processed for caspase-3 and -9 activity. Data are the means of at least three independent experiments. Error bars indicate SD.

These latter data are consistent with the induction of cancer cell death through apoptosis. It is well known that, in addition to determine a profound effect on maintaining redox state, TrxR system has a direct interaction with the apoptotic pathway through binding of Trx to ASK1, a member of the MAPKKK family [33,35]. Inhibition of TrxR system therefore results in activation of Apoptosis signal-regulating kinase 1(ASK1) and stimulation of apoptosis. As it was recently demonstrated that Trx directly regulate apoptosis by interacting with procaspase-3 and -9 [35,36], we also monitored the ability of complexes **5** and **10** to activate initiator caspase-9 and effector caspase-3. Cells were incubated for 24 or 48 h with IC_50_ of compounds **5** or **10**, with or without pre-treatment with broad-spectrum caspase inhibitor zVAD, and processed caspase-3 and -9 activities were monitored fluorimetrically by means of specific fluorogenic substrates. As reported in Figure 8, complexes **5** and **10** provoked a time-dependent activation of caspase-3. Caspase-9 activation, however, was already maximized following 24 h of treatment with both silver(I) complexes. In particular, by treating 2008 cells with IC_50_ concentration of **5** and **10**, a 3.3- and 2.6-fold enhancement in caspase-9 activity, compared to control cells, has been recorded, respectively.

## 3. Materials and Methods

All reactions were routinely performed under a dry nitrogen atmosphere, using standard Schlenk techniques. Commercially available substances were of reagent grade and used without further purification. The Ag(I) precursor [Ag(CH_3_CN)_4_][BF_4_] was prepared by reaction of Ag_2_O with HBF_4_ in acetonitrile. The ligands PTA [37], DAPTA [38], PTA-SO_2_ [39] and K[HB(pz)_3_] were synthesized by published methods. Elemental analyses were performed on a Carlo Erba 1106 Elemental Analyzer. ^1^H, ^31^P and ^13^C spectra were recorded on a Bruker AMX-300 instrument (300.13 MHz for ^1^H, 121.41 MHz for ^31^P, 75.47 MHz for ^13^C), using SiMe_4_ as internal reference (^1^H and ^13^C) and 85% aqueous H_3_PO_4_ as external reference (^31^P). FT IR spectra were recorded on a Mattson 3030 Fourier transform spectrometer in the range 4000–400 cm^−1^ in KBr pellets. Mass spectra have been recorded by an electrospray LCQ Thermo Finnigan mass spectrometer.

The purities of compounds used for biological activity determination, were checked by elemental analysis and were found to be ≥95%.

### 3.1. Synthesis of Heteroleptic Silver Complexes

All of the silver complexes were synthesized according to the procedure here detailed for [HB(pz)_3_]Ag(PPh_3_)]. In degassed acetonitrile (15 mL) under stirring, an equimolar amount of [Ag(MeCN)_4_][BF_4_] (108 mg, 0.3 mmol), PPh_3_ (79 mg, 0.3 mmol) and K[HB(pz)_3_] (76 mg, 0.3 mmol) were dissolved at room temperature. After 3h, the solvent was eliminated and the residue treated with chloroform. The obtained suspension was filtered, the solvent evaporated and the resulting white product washed with diethyl ether and dried under vacuum. Different attempts were performed to crystallize the resulting powders, but only compound **5** gave crystals suitable for X-ray characterization. Structural data (data not shown) confirmed what already reported by C. Santini et al. in ref. [21]. Representative IR, NMR and mass spectra are reported in the Supplementary Materials.

**[HB(pz)_3_]Ag(PTA) (1).** Slightly grey product. Yield: 67%. ^1^H NMR (CDCl_3_): δ (ppm) = 4.37 (s, 6H, PC*H*_2_N), 4.63 (d+d, 6H, NC*H*_2_N), 6.14 (t, 3H, C*H*_pz_); 7.45 (d, 3H, C*H*_pz_), 7.63 (d, 3H, C*H*_pz_). ^31^P{H} NMR (CDCl_3,_ 293 K): δ (ppm) = −80.7 d (^1^J_P-Ag_ 605 Hz). ^13^C{H} NMR (CDCl_3_): 141.4 (s, *C*H_pz_), 135.6 (s, *C*H_pz_), 104.0 (s, *C*H_pz_), 74.1 (N-*C*H_2_-N), 52.9 (P-*C*H_2_-N). ^1^H NMR (DMSO-d_6_): δ (ppm) 4.49 (d, 6H, PC*H*_2_N), 4.58 (q, 6H, NC*H*_2_N), 6.14 (t, 3H, C*H*_pz_), 7.49 (d, 3H, C*H*_pz_), 7.70 (d, 3H, C*H*_pz_). ^31^P{H} NMR (DMSO-d6): δ (ppm) −80.0 (bs). IR (KBr, cm^−1^) ν 2441 m (B-H); 1396 m (B-N). ESI-MS (MeOH) (*m*/*z* assignment, intensity %): 478.0 ([M + H]^+^, 100); 499.9 ([M + Na]^+^, 10); 742.9 ([M + Ag(PTA)]^+^, 40). Anal. Calcd for AgPBN_9_C_15_H_22_ C 37.87, H 4.64, N 26.37. Found: C 38.01, H 4.76, N 25.97.

**[HB(pz)_3_]Ag(DAPTA) (2).** Yellowish product. Yield: 60%. ^1^H NMR (CDCl_3_): δ (ppm) = 2.04 (s, 6H, C*H*_3_), 3.45 (ddd, 1H, H^a2^), 3.95 (d, 2H, H^b^), 4.16 (d, 1H, H^e1^), 4.30 (ddd, 1H, H^c2^), 4.65 (d, 1H, H^c1^), 4.75 (d, 1H, H^d^), 5.04 (d, 1H, H^d^), 5.69 (d, 1H, H^a1^), 5.88 (d, 1H, H^e2^), 6.21 (t, 3H, C*H*_pz_), 7.53(d, 3H, C*H*_pz_), 7.67(d, 3H, C*H*_pz_). ^31^P{H} NMR (CDCl_3_): δ (ppm) = −55.31 (bs). ^13^C{H} NMR (CDCl_3_):170.0 (C=O), 141.6 (*C*H_pz_), 135.8 (*C*H_pz_), 104.3 (*C*H_pz_), 67.9 (N-*C*^5^-N), 62.7 (N-*C*^4^-N), 49.5 (P-*C*^2^-N), 44.8 (P-*C*^3^-N), 38.8 (P-*C*^1^-N), 21.1 (s, *C*^8^H_3_), 21.7 (s, *C*^9^H_3_). ^1^H NMR (DMSO-d6): δ (ppm) = 2.03 (m, 6H, C*H*_3_), 3.80 (ddd, 1H, CH-a2), 4.5 (bs, 2H, CH-b), 4.23 (d, 1H, CH-e1), 4.35 (ddd, 1H, CH-c2), 4.80 (m, 2H, CH-c1 + CH-d), 4.97 (d, 1H, CH-d), 5.48 (d, 1H, CH-a1), 5.64 (d, 1H, CH-e2), 6.16 (t, 3H, C*H*_pz_), 7.55(d, 3H, C*H*_pz_), 7.72(d, 3H, C*H*_pz_) ^31^P{H} NMR (DMSO-d6, 298 K): δ (ppm) 53.6 (d) (^1^J_P-Ag_ 605 Hz). IR (KBr, cm^−1^) ν 2429m (B-H); 1639s (C=O). ESI-MS (MeOH) (*m*/*z* assignment, intensity %): 549.9 ([M + H]^+^, 60); 573.0 ([M + Na]^+^,18); 1070.7 ([Ag_3_(HBpz_3_)_3_ + Ag]^+^, 100). Anal Calcd for AgPBO_2_N_9_C_18_H_26_ C 39.30, H 4.76, N 22.92. Found: C 39.91, H 4.97, N 22.56. DAPTA numbering scheme is reported in Supplementary Materials (Figure S1).

**[HB(pz)_3_]Ag(PTA-SO_2_) (3).** Grey product. Yield 50%. ^1^H NMR (CDCl_3_): δ (ppm) = 4.24 (s, 2H, PCH_2_N), 4.64 (m, 1H), 4.70 (m, 1H), 4.99 (d, 2H), 5.18 (m, 4H), 6.16 (t, 3H, CH_pz_), 7.44 (d, 3H, CH_pz_), 7.67 (d, 3H, CH_pz_). ^31^P {H} NMR (CDCl_3_): δ (ppm) = −92.6 (bs). ^13^C {H} NMR (CDCl_3_): δ (ppm) 141.4 (s, CH_pz_), 135.8 (s, CH_pz_), 104.2 (s, CH_pz_), 73.59 (s, N-CH_2_), 52.37 (s, P-CH_2_). ^1^H NMR (DMSO-d_6_): δ (ppm) = 4.34 (d, 2H, PCH_2_N), 4.84 (m, 4H, PCH2N), 5.06 (d, 2H, NCHN), 5.29 (d, 2H, NCHN), 6.11 (s, 3H, CH_pz_), 7.50 (s, 3H, CH_pz_), 7.68 (s, 3H, CH_pz_). ^31^P{H} NMR (DMSO-d_6_): δ (ppm) = −89.6 (s). IR (KBr, cm^−1^) ν 2453m (B-H); 1384s (SO_2_); 1187s (SO_2_). ESI-MS (MeOH) (*m*/*z* assignment, intensity %): 528.0 ([M + H]^+^, 50); 749. 1 ({[Ag(HBPz_3_)]_2_ + Ag}^+^, 60); 1070.8 ({[Ag(HBPz_3_)]_3_ + Ag}^+^, 100). Anal Calcd for AgPSO_2_BN_9_C_14_H_20_ C 31.84, H 3.82, N 23.87. Found: C 32.05, H 3.67, N 23.56.

**[HB(pz)_3_]Ag(PCN) (4).** Due to its low solubility in CHCl_3_, 4 was recovered from the reaction mixture by evaporating the solvent, and washing the residue with water (to eliminate KBF_4_) and diethyl ether. The resulting beige powder was dried under vacuum. Yield 65%. ^1^H NMR (DMSO-d_6_): δ (ppm) 2.33 (m, 6H, CH_2_CN), 2.84 (m, 6H CH_2_P), 6.18 (t, 3H, CH_pz_), 7.59 (d, 3H, CH_pz_), 7.74 (d, 3H, CH_pz_). ^31^P{H} NMR (DMSO-d_6_): δ (ppm) 8.0 (d) (J_P-Ag_ 664 Hz). ^13^C{H} NMR (DMSO): δ (ppm) 14.4 (d, CH_2_-P), 21.3 (d, CH_2_-N), 104.7 (s, CH_pz_), 120.8 (d, CN), 136.0 (s, CH_pz_), 141.6 (s, CH_pz_)). ^31^P {H} NMR (CDCl_3_): δ (ppm) 15.1 d(J_P-Ag_ 635 Hz) ESI-MS (MeOH) (*m*/*z* assignment, intensity %): 513.9 ([M + H]^+^, 100); 538.0 ([M + Na]^+^, 20). IR (KBr, cm^−1^) 2464 w (B-H); 2254 w (CN); 1398 m (B-N). AgPBN_9_C_18_H_22_ C 42.05, H 4.31, N 24.52; found: C 42.34, H 4.05, N 24.91.

**[HB(pz)_3_]Ag(PPh_3_) (5).** Characterization of compound **5** is in agreement with data already reported in the literature [21]. White powder. Yield 47%. ^1^H NMR (CDCl_3_): δ (ppm) = 6.13 (t, 3H, CH_pz_), 7.42 (d, 3H, CH_pz_), 7.49 (m, 9H, H_Ph_), 7.60 (m, 6H, H_Ph_), 7.70 (d, 3H, CH_pz_). ^31^P{H} NMR (CDCl_3,_ 298 k): δ (ppm) = 17.50 (dd, ^1^J(P, ^107^Ag) = 613 Hz, ^1^J(P, ^109^Ag) = 692 Hz). ^13^C{H}NMR (CDCl_3_): δ (ppm) 141.4 (s, CH_pz_), 135.5 (s, CH_pz_), 134.0, 132.4, 130.7, 129.3 (d), 104.1 (s, CH_pz_).^1^H NMR (DMSO-d6): δ (ppm) = 6.18 (t, 3H, CH_Pz_), 7.42 (d, 3H, CH_Pz_), 7.59 (m, 15H, H_Ph_), 7.78 (d, 3H, CH_Pz_). ^31^P{H} NMR (DMSO-d6): δ (ppm) = 15.9 (d).^. 13^C{H}NMR (DMSO-d6): δ (ppm) 141.3(s, CH_pz_), 136.2(s, CH_pz_), 134.5, 134.30, 131.8, 130.4 (d), 104.9 (s, CH_pz_). IR (KBr, cm^−1^) ν 2443 m (B-H); 1394 m (B-N). ESI-MS (MeOH) (*m*/*z* assignment, intensity %): 953 ([M + Ag(PPh_3_)]^+^, 100); 633 ([Ag(PPh_3_)_2_]^+^, 25); 585 ([M + H]^+^, 8). Anal. Calcd. for AgPBN_6_C_27_H_25_; C 55.61, H 4.32, N 14.41; found: C 55.95, H 4.04, N 14.72.

### 3.2. Synthesis and Characterization of Homoleptic Phosphino Silver Complexes

A general procedure was employed for the synthesis of silver compounds **6**–**10**. To an acetonitrile solution (10 mL) of AgBF_4_ (20 mg, 0.1 mmol) a stoichiometric amount of phosphine ligand (0.4 mmol) was added at room temperature. After 3 h the volume of the reaction mixture was reduced by solvent evaporation to ca 2 mL. By addition of diethyl ether, a white precipitate was recovered by filtration and dried under vacuum. Characterization of compounds **6**, **9,** and **10** is in agreement with data already reported in the literature for analogous complexes differing only for the anion, namely [Ag(PTA)_4_]PF_6_ [9,26], [Ag(PCN)_2_]NO_3_ [22], [Ag(PPh_3_)_4_]PF_6_ [40] and [Ag(PPh_3_)_4_]ClO_4_ [41].

**[Ag(PTA)_4_]BF_4_ (6).** White water soluble product. Yield: 85%. ^1^H NMR (D_2_O): δ (ppm) = 4.07 (d, 6H, C*H*_2_P), 4.51 (AB q, 6H, NC*H*_2_N). ^31^P{H} NMR (D_2_O): δ (ppm) = −83.9(d). ESI-MS(+) in MeOH (*m*/*z* assignment, % intensity): 421 ([Ag(PTA)_2_]^+^, 100). Anal. Calcd. for AgP_4_N_12_C_24_H_48_BF_4_^.^3H_2_O: C 32.87, H 6.21, N 19.18%. Found: C 33.01, H 5.98, N 19.46%.

**[Ag(DAPTA)_4_]BF_4_ (7).** White water-soluble product. Yield: 70%. ^1^H NMR (D_2_O): δ (ppm) 5.59 (d, 1H, H^e2^), 5.40 (d, 1H, H^d1^), 5.10 (d, 1H, H^a1^), 4.60 (d, 1H, H^d2^), 4.60 (d, 1H, H^c1^), 4.18 (d, 1H, H^e1^), 4.14 (dd, 1H, H^c2^), 3.76 (d, 2H, H ^b1+b2^), 3.54 (dd, 1H, H^a2^), 2.04 (d, 6H, CH_3_). ^31^P{H} NMR (D_2_O): δ (ppm) −61.0 (s). ESI-MS(+) in MeOH (*m*/*z* assignment, % intensity): 565.4 ([Ag(DAPTA)_2_]^+^, 100), 481.3 ([(DAPTA)_2_ + Na]^+^, 92), 252.3 ([DAPTA + Na]^+^, 43). Anal. Calcd. for AgC_36_H_64_N_12_O_8_P_4_BF_4_: C 38.90, H 5.80, N 15.12%. Found: C 38.36, H 5.87, N 15.34%.

**[Ag(PTA-SO_2_)_4_]BF_4_ (8).** White powder. Yield: 65%. ^1^H NMR (DMSO): δ (ppm) 5.1 (d, 2H, NC*H_2_*N), 4.78 (d, 2H, NC*H*_2_N), 4.58 (dd, 2H, PC*H*_2_N), 4.65 (dd, 2H, PC*H*_2_N), 3.96 (d, 2H, PC*H*_2_N). ^31^P{H} NMR (DMSO): δ(ppm) −107.0. ESI-MS(+) (*m*/*z* assignment, % intensity) in DMSO/MeOH: 392.2 ([Ag(PTA-SO_2_)(DMSO)]^+^, 100). Anal. Calcd for AgP_4_S_4_O_8_N_12_H_40_C_20_BF_4_. C 23.47, H 3.94, N 16.42%. Found: C 23.79, H 3.94, N 16.15%

**[Ag(PCN)_2_]BF_4_ (9).** White powder. Yield: 85%. ^1^H NMR (DMSO): δ (ppm) 2.80 (bs, 2H), 2.18 (bs, 2H) ^1^H NMR (CDCl_3_): δ (ppm) 2.62 (dt, 2H), 1.96 (t, 2H). ^31^P{H} NMR (DMSO): δ(ppm) −2.08 bs. ESI-MS(+) (*m*/*z* assignment, % intensity) in DMSO/MeOH: 493 ([Ag(PCN)_2_]^+^, 100). Anal. Calcd for AgP_2_C_18_H_24_N_6_BF_4_: C 37.21, H 4.16, N 14.46% Found: C 37.76, H 4.69, N 14.12%.

**[Ag(PPh_3_)_4_]BF_4_ (10).** White powder. Yield: 85%. ^1^H NMR (CDCl_3_): δ (ppm) 7.39 (t, 1H), 7.18 (t, 2H), 7.08 (t, 2H). ^1^H NMR (DMSO): δ (ppm) 7.45 (t, 1H), 7.35 (t, 2H), 7.22 (t, 2H). ^31^P{H} NMR (CDCl_3_): δ(ppm) 7.70 s. ^31^P{H} NMR (DMSO): δ(ppm) 3.69 s. ESI-MS(+) in CHCl_3_/MeOH (*m*/*z* assignment, % intensity): 633 ([Ag(PPh_3_)_2_]^+^, 100). Anal. Calcd. for AgP_4_C_72_H_60_BF_4_ C 69.5, H 4.86%. Found: C 69.12, H 4.66%.

### 3.3. Experiments with Cultured Human Cancer Cells

Heteroleptic (**1**–**5**) and homoleptic (**8**–**10**) Ag(I) complexes were dissolved in DMSO just before the experiment, and a calculated amount of drug solution was added to the cell growth medium to a final solvent concentration of 0.5%, which had no detectable effects on cell viability.

Homoleptic compounds **6** and **7** as well as cisplatin were dissolved in 0.9% sodium chloride solution. MTT (3-(4,5-dimethylthiazol-2-yl)-2,5-diphenyltetrazolium bromide) and cisplatin were obtained from Sigma Chemical Co, St. Louis, USA.

#### 3.3.1. Cell Cultures

Human lung (A549), breast (MCF-7), colon (HCT-15), and pancreatic (BxPC3) carcinoma cell lines along with melanoma (A375) cells were obtained by American Type Culture Collection (ATCC, Rockville, MD, USA). A431 are human cervical carcinoma cells kindly provided by Professor F. Zunino (Division of Experimental Oncology B, Istituto Nazionale dei Tumori, Milan, Italy). The 2008 cells and cisplatin-resistant variant, C13*, are human ovarian adenocarcinoma cell lines that were kindly provided by Professor G. Marverti (Department of Biomedical Science of Modena University, Italy). Cell lines were maintained in the logarithmic phase at 37 °C in a 5% carbon dioxide atmosphere using the following culture media containing 10% fetal calf serum (EuroClone, Milan, Italy), antibiotics (50 units/mL penicillin and 50 μg/mL streptomycin) and 2 mM l-glutamine: (i) RPMI-1640 medium (EuroClone) for MCF-7, A431, BxPC3, 2008 and C13* cells; (ii) F-12 HAM’S (Sigma Chemical Co.) for A549 cells; (iii) DMEM for A375 cells.

#### 3.3.2. MTT Assay

The growth inhibitory effect towards tumor cells was evaluated by means of MTT assay [42]. Briefly, 3–8 × 10^3^ cells/well, dependent upon the growth characteristics of the cell line, were seeded in 96-well microplates in growth medium (100 μL). After 24 h, the medium was removed and replaced with a fresh one containing the compound to be studied at the appropriate concentration. Triplicate cultures were established for each treatment. After 72 h, each well was treated with 10 μL of a 5 mg/mL MTT saline solution, and following 5 h of incubation, 100 μL of a sodium dodecyl sulfate (SDS) solution in HCl 0.01 M were added. After an overnight incubation, cell growth inhibition was detected by measuring the absorbance of each well at 570 nm using a Bio-Rad 680 microplate reader. Mean absorbance for each drug dose was expressed as a percentage of the control untreated well absorbance and plotted vs drug concentration. IC_50_ values, the drug concentrations that reduce the mean absorbance at 570 nm to 50% of those in the untreated control wells, were calculated by the four parameter logistic (4-PL) model. Evaluation was based on means from at least four independent experiments.

### 3.4. In Vitro TrxR1 Inhibition

The assay was performed in 0.2 M Na–K-phosphate buffer pH 7.4, containing 5 mM EDTA, 0.250 mM nicotinamide adenine dinucleotide phosphate (NADPH) and 75 nmol of TrxR1 (IMCO, Sweden). The silver(I) complexes as well as auranofin were pre-incubated for 5 min at room temperature; the reaction started with 1 mM DTNB (5,5′-dithiobis(2-nitrobenzoic acid)), and the increase of absorbance was monitored at 412 nm over 5 min at 25 °C. Enzyme activity was calculated taking into account that 1 mol of NADPH yields 2 mol of CNTP anion (carboxy-nitro-thiophenol, reduced DTNB).

### 3.5. Reactive Oxygen Species (ROS) Production

The production of ROS was measured in 2008 cells (10^4^ per well) grown for 24 h in a 96-well plate in RPMI medium without phenol red (Sigma Chemical Co.). Cells were then washed with PBS and loaded with 10 μM 5-(and-6)-chloromethyl-2′,7′-dichlorodihydrofluorescein diacetate acetyl ester (CM–H_2_DCFDA) (Molecular Probes-Invitrogen, Eugene, OR) for 25 min, in the dark. Afterwards, cells were washed with PBS and incubated with increasing concentrations of tested compounds. Fluorescence increase was estimated utilizing the wavelengths of 485 nm (excitation) and 527 nm (emission) in a Fluoroskan Ascent FL (Labsystems, Finland) plate reader. Antimycin (3 μM, Sigma Chemical Co), a potent inhibitor of Complex III in the electron transport chain, and auranofin were used as positive controls.

### 3.6. Quantification of Thiols

The 2008 cells (2 × 10^5^) were seeded in a six-well plate in growth medium (4 mL). After 24 h, cells were incubated for 24 h with increasing concentrations of tested compounds. Subsequently, the thiol content was measured as previously described [43].

### 3.7. Mitochondrial Membrane Potential (*ΔΨ*)

The ΔΨ was assayed using the Mito-ID^®^ Membrane Potential Kit according to the manufacturer’s instructions (Enzo Life Sciences, Farmingdale, NY). Briefly, 2008 cells (8 × 10^3^ per well) were seeded in 96-well plates; after 24 h, cells were washed with PBS and loaded with Mito-ID Detection Reagent for 30 min at 37 °C in the dark. Afterwards, cells were incubated with increasing concentrations of tested complexes. Fluorescence intensity was estimated using a plate reader (Fluoroskan Ascent FL, Labsystems, Finland) at 490 (excitation) and 590 nm (emission). Carbonyl cyanide m-chlorophenyl hydrazone (CCCP, 4 μM), a chemical inhibitor of the oxidative phosphorylation, was used as positive control.

### 3.8. Caspase-3 and -9 Activation

The 2008 cells (1 × 10^6^) were treated for 24 h with the IC_50_ doses of tested compounds, harvested and homogenized in a lysis buffer [1% Triton X-100, 320 nM sucrose, 5 mM EDTA, 10 mM Tris–HCl and 2 mM DTT (1,4-dithio-DL-threitol) buffer (pH 7.6)]. Protein aliquots (100 μg) were stained at 37 °C for 60 min with fluorescent caspase-3 (N-Acetyl-Asp-Glu-Val-Asp-AMC, AMC = 7-amino-4-methylcoumarin) or caspase-9 (Ac-Leu-Glu-His-Asp-AMC) substrate (Sigma Co.). Substrate hydrolysis was measured after 60 min by monitoring the release of AMC using a spectrofluorometer (excitation at 370 nm, emission at 460 nm).

### 3.9. Transmission Electron Microscopy (TEM) Analyses

About 10^6^ 2008 cells were seeded in 24-well plates and, after 24 h incubation, were treated with the tested compounds and incubated for additional 24 h. Cells were then washed with cold PBS, harvested and directly fixed with 1.5% glutaraldehyde buffer with 0.2 M sodium cacodylate, pH 7.4. After washing with buffer and postfixation with 1% OsO_4_ in 0.2 M cacodylate buffer, specimens were dehydrated and embedded in epoxy resin (Epon Araldite). Sagittal serial sections (1 μm) were counterstained with toluidine blue; thin sections (90 nm) were given contrast by staining with uranyl acetate and lead citrate. Micrographs were taken with a Hitachi H-600 electron microscope (Hitachi, Tokyo, Japan) operating at 75 kV. All photos were typeset in Corel Draw 11.

### 3.10. Comet Assay

About 4 × 10^4^ 2008 cells were seeded in 25 cm^2^ flasks in growth medium (6 mL). After 24 h, cells were incubated for 3h with 2.5µM of tested compounds. Cells were washed twice with cold PBS, harvested, centrifuged, and DNA fragmentation was measured by the alkaline comet assay. Low melting point agarose, 300 μL (Trevigen Inc., Gaithersburg, MD, USA) was heated to 37 °C and combined with 2 × 10^5^ cells per mL cell suspension. Each well of a 20-well CometSlide was filled with 30 μL of the cell/agarose suspension. The slides were placed in a 4 °C refrigerator in the dark for 15 min to solidify. Slides were then immersed in 50 mL of pre-chilled lysis solution containing Trizma base, Triton X-100, DMSO and left at 4 °C for 30 min to facilitate cell membrane and histone removal. After draining excess liquid, the slides were transferred to 50 mL of freshly prepared (same day) alkaline DNA unwinding solution, (200 mmol/L NaOH, 1 mmol/L EDTA, pH > 13) and incubated at room temperature in the dark for 20 min. After the unwinding step, electrophoresis was performed at 21 V for 30 min. Slides were then rinsed with distilled water and fixed 5 min in 70% ethanol. Slides were dried and stained 5 min at 4 °C with SYBR Green I (Trevigen, Inc.) diluted 1:10000 in 10 mmol/L Tris pH 7.5, 1 mmol/L EDTA, drained to remove excess staining solution and thoroughly dried at room temperature in the dark. Micrographs were taken with a Zeiss LSM-800 confocal microscope (Zeiss, Oberkochen, Germany). All photos were typeset in Zen 2.3 system (Zeiss, Oberkochen, Germany).

### 3.11. Statistical Analysis

All values are the means ± SD of no less than three measurements starting from three different cell cultures. Multiple comparisons were made by ANOVA followed by the Tukey–Kramer multiple comparison test (* *p* < 0.05, ** *p* < 0.01), using GraphPad software.

## 4. Conclusions

Two series of phosphino Ag(I) compounds, namely heteroleptic, ‘3+1’-type [(HB(pz)_3_]Ag(PR_3_) complexes **1**–**5**, and homoleptic, ‘AgP_4_’-type [Ag(PR_3_)_4_]BF_4_ complexes **6**–**10** have been prepared starting from [Ag(MeCN)_4_]BF_4_ and AgBF_4_ precursors, respectively. Both series were evaluated in vitro for their cytotoxic potential against a wide panel of human cancer cells derived from solid tumors, and endowed with different platinum drug sensitivity. Heteroleptic complexes **1**–**5** were generally more effective (IC_50_ in the range 1.52–10.32 μM) than the corresponding homoleptic derivatives **6**–**10** (IC_50_ in the range 1.98–17.73 μM). In both series, cytotoxicity was function of phosphine lipophilicity following the trend PPh_3_ > PCN ≥ PTA-SO_2_ > PTA ≥ DAPTA, the most active compounds being [HB(pz)_3_]Ag(PPh_3_), **5** (IC_50_ in the range 1.52–4.44 μM), and [Ag(PPh_3_)_4_]BF_4,_ **10** (IC_50_ in the range 1.98–5.12 μM).

All tested complexes were able to overcome Pt(II) drug and MDR resistance thus confirming the usefulness of coordinating silver(I) ions with ligands containing P-donor atoms in the development of anticancer metallodrugs alternative to platinum drugs. By mechanistic studies both heteroleptic and homoleptic Ag(I) complexes were found to selectively inhibit mammalian TrxR in the low nanomolar range. Towards isolated enzyme, as well as in whole cells, Ag(I) complexes comprising lipophilic phosphine ligands were more potent than the hydrophilic monocationic derivatives in inhibiting TrxR activity suggesting that the polar character of the overall complex may interfere with enzyme binding. In particular, the most drastic inhibition of TrxR, both on the purified enzyme and on cell extracts, was detected with [HB(pz)_3_]Ag(PPh_3_) (**5**) and [Ag(PPh_3_)_4_]BF_4_ (**10**). These findings, in accordance with other reports, point out TrxR as a leading molecular target for various classes of silver(I) compounds. In contrast, none of the tested Ag(I) complexes were able to inhibit Gpx, the other major redox-regulating selenoenzyme, demonstrating that enzyme structure-specific small molecule interactions, have a significant influence over the inherent reactivity of the Sec residue. Actually, unlike the flexible C-terminus active site of TrxR, the active site of Gpx locates at the N-terminal ends of long α-helices, surrounded by aromatic side-chains. This structural context may therefore protect Sec from phosphino Ag(I) species. The marked TrxR inhibitory effect induced by **5** and **10** in human 2008 ovarian cancer cells was accompanied by a significant decrease of the total sulfhydryl groups and overproduction of ROS. The latter effect resulted in a strong decrease in the mitochondrial membrane potential and activation of caspase 3, thus leading cancer cells to apoptosis, as evidenced by TEM analysis. Noteworthy, only for mixed-ligand complexes, and in particular for complex **5**, a marked DNA damaging ability has been evidenced in both cell-free systems and whole cancer cells. The presence of the HB(pz)_3_ ligand in the metal coordination sphere, enabling Ag(I) species to interact with an additional cellular target, may account for the overall higher cytotoxicity evidenced for heteroleptic phosphine Ag(I) complexes compared to homoleptic ones.

In conclusion, our results confirm the importance of the choice of coordinating ligands and their combination in affecting the biological behavior of the corresponding silver(I) complexes. The ligand(s) assembly around the silver(I) ion is a determining factor not only in terms of hydrophilic–lipophilic balance, but also in terms of biological mechanism of action responsible for cancer cell growth inhibition.

## Figures and Tables

**Table 1:** ^31^P NMR parameters. Δδ(^31^P )(ppm) = δ (^31^P _complex_) − δ(^31^P _ligand_) (ppm) * doublet, ** doublet of doublets. In brackets ^1^J(Ag-P) in Hz.

	Δδ(^31^P ) ppm (CDCl_3_)	Δδ(^31^P ) ppm (DMSO)
[HB(pz)_3_]Ag(PTA) (1)	20.5 * (605)	23.8
[HB(pz)_3_]Ag(DAPTA) (2)	23.1	24.7 * (605)
[HB(pz)_3_]Ag(PTA-SO_2_)(3)	24.4	26.6
[HB(pz)_3_]Ag(PCN) (4)	35.0 *(635)	31.9 * (664)
[HB(pz)_3_]Ag(PPh_3_) (5)	22.8 ** (^1^J(P, ^107^Ag) = 613 Hz, ^1^J(P, ^109^Ag) = 692 Hz)	20.8 * (ca 680)

**Table 2:** Cytotoxicity assays. Cells (3–8 × 10^3^·mL^−1^) were treated for 72 h with increasing concentrations of tested compounds. Cytotoxicity was assessed by MTT test. IC_50_ values were calculated by a four parameter logistic model (*p* < 0.05). S.D. = standard deviation.

Compound	IC_50_ (µM) ± S.D.					
	BxPC3	HCT-15	MCF-7	A431	A375	A549
**[HB(pz)_3_]Ag** **(PTA) (1)**	6.6 ± 2.8	8.5 ± 1.8	6.5 ± 3.1	10.3 ± 1.0	8.1 ± 1.0	5.1 ± 1.2
**[HB(pz)_3_]Ag** **(DAPTA) (2)**	7.6 ± 2.3	7.6 ± 2.0	7.4 ± 4.1	6.7 ± 1.3	9.2 ± 0.1	7.5 ± 0.1
**[HB(pz)_3_]Ag** **(PTA-SO_2_) (3)**	5.1 ± 1.4	7.4 ± 2.2	5.1 ± 1.7	4.5 ± 1.2	5.2 ± 2.1	8.3 ± 2.0
**[HB(pz)_3_]Ag** **(PCN) (4)**	5.6 ± 1.9	3.3 ± 1.2	5.1 ± 1.4	2.9 ± 1.0	8.9 ± 2.5	4.1 ± 1.0
**[HB(pz)_3_]Ag** **(PPh_3_) (5)**	4.4 ± 1.6	2.1 ± 0.6	2.9 ± 1.0	1.5 ± 0.5	2.2 ± 0.7	2.1 ± 0.6
**[Ag** **(PTA)_4_]BF_4_ (6)**	13.2 ± 2.2	10.2 ± 3.1	11.5 ± 1.8	9.3 ± 2.2	8.2 ± 4.1	10.2 ± 2.5
**[Ag** **(DAPTA)_4_]BF_4_ (7)**	7.5 ± 3.2	16.2 ± 7.2	11.3 ± 2.2	11.2 ± 2.1	9.1 ± 2.2	8.5 ± 3.3
**[Ag** **(PTA-SO_2_)_4_]BF_4_ (8)**	5.3 ± 5.5	17.7 ± 4.6	12.2 ± 1.6	4.1 ± 1.2	6.3 ± 3.3	13.2 ± 2.5
**[Ag** **(PCN)_2_]BF_4_ (9)**	3.1 ± 1.6	3.6 ± 1.1	4.0 ± 1.0	2.9 ± 0.5	5.3 ± 1.1	5.5 ± 1.6
**[Ag** **(PPh_3_)_4_]BF_4_ (10)**	3.1 ± 0.8	3.4 ± 1.0	5.1 ± 1.2	2.0 ± 0.5	3.2 ± 1.1	3.0 ± 1.0
**PTA**	>100	>100	>100	>100	>100	>100
**DAPTA**	89.5 ± 4.1	67.1 ± 3.1	85.3 ± 4.0	>100	>100	>100
**PCN**	>100	>100	>100	>100	>100	>100
**PPh_3_**	62.2 ± 2.2	54.1 ± 4.2	34.2 ± 3.4	47.5 ± 1.1	52.3 ± 2.5	30.1 ± 2.1
**PTA-SO_2_**	>100	>100	69.5 ± 3.4	56.0 ± 3.5	>100	>100
**Na(HBpz_3_)**	97.4 ± 2.2	77.6 ± 2.0	>100	40.4 ± 1.4	66.9 ± 2.3	>100
**Cisplatin**	10.2 ± 1.7	15.5 ± 2.5	7.6 ± 3.0	2.1 ± 0.4	4.0 ± 1.0	8.4 ± 0.9

**Table 3:** Cross-resistance profiles. Cells (3 × 10^3^·mL^−1^) were treated for 72 h with increasing concentrations of tested compounds. Cytotoxicity was assessed by MTT test. IC_50_ values were calculated by a four parameter logistic model (*p* < 0.05). RF = IC_50_ resistant/IC_50_ parental cell lines. S.D. = standard deviation.

Compound	IC_50_ (µM) ± D.S.		
	2008	C13*	RF
**[HB(pz)_3_]Ag(PTA) (1)**	6.7 ± 3.2	7.4 ± 1.5	1.1
**[HB(pz)_3_]Ag(DAPTA) (2)**	8.9 ± 4.2	8.4 ± 1.3	0.9
**[HB(pz)_3_]Ag(PTA-SO_2_) (3)**	6.3 ± 1.1	5.6 ± 1.4	0.9
**[HB(pz)_3_]Ag(PCN) (4)**	7.8 ± 2.4	6.5 ± 1.8	0.9
**[HB(pz)_3_]Ag(PPh_3_) (5)**	2.02 ± 1.2	2.1 ± 1.5	1.0
**[Ag(PTA)_4_]BF_4_ (6)**	9.23 ± 2.0	10.9 ± 3.5	1.2
**[Ag(DAPTA)_4_]BF_4_ (7)**	10.3 ± 3.1	13.4 ± 2.4	1.3
**[Ag(PTA-SO_2_)_4_]BF_4_ (8)**	13.2 ± 2.5	16.3 ± 3.3	1.2
**[Ag(PCN)_2_]BF_4_ (9)**	3.1 ± 0.6	4.1 ± 1.1	1.3
**[Ag(PPh_3_)_4_]BF_4_ (10)**	4.2 ± 0.8	6.1 ± 1.3	1.4
**Cisplatin**	2.3 ± 1.1	21.5 ± 3.0	9.5

**Table 4:** Thioredoxin reductase 1 activity was assayed by measuring NADPH-dependent reduction of DTNB at 412 nm; IC_50_ values were calculated by a four parameter logistic model (*p* < 0.05).

Compound	IC_50_ (nM)
**[HB(pz)_3_]Ag(PTA) (1)**	2.8
**[HB(pz)_3_]Ag(DAPTA) (2)**	3.5
**[HB(pz)_3_]Ag (PTA-SO_2_) (3)**	2.6
**[HB(pz)_3_]Ag(PCN) (4)**	2.7
**[HB(pz)_3_]Ag(PPh_3_) (5)**	3.3
**[Ag(PTA)_4_]BF_4_ (6)**	9.3
**[Ag(DAPTA)_4_]BF_4_ (7)**	7.2
**[Ag(PTA-SO_2_)_4_]BF_4_ (8)**	16.8
**[Ag(PCN)_2_]BF_4_ (9)**	2.4
**[Ag(PPh_3_)_4_]BF_4_ (10)**	3.2
**AgNO_3_**	2.4
**Auranofin**	0.8

## Supplementary Materials

The following are available online, Figure S1: Numbering scheme of DAPTA ligand. Figure S2: IR spectra in KBr of [HB(pz)_3_]Ag(PTA) (**1**) and [HB(pz)_3_]Ag(DAPTA) (**2**). Figure S3: IR spectra in KBr of [HB(pz)_3_]Ag(PTA-SO_2_) (3) and [HB(pz)_3_]Ag(PCN) (4). Figure S4: ^1^H NMR spectra of [HB(pz)_3_]Ag(PTA) (**1**) in DMSO and of [HB(pz)_3_]Ag(DAPTA) (**2**) in CDCl_3_. Figure S5: ^1^H NMR of [HB(pz)_3_]Ag(PTA-SO_2_) (**3**) and [HB(pz)_3_]Ag(PCN) (**4**) in CDCl_3._ Figure S6: ^31^P NMR spectra of [HB(pz)_3_]Ag(PTA) (**1**) and [HB(pz)_3_]Ag(DAPTA)(**2**) in different solvents at room temperature. Figure S7: NMR characterization of [HB(pz)_3_]Ag(PTA-SO_2_) (3) in CDCl_3_: ^31^P and ^13^C. Figure S8: ^31^P NMR spectra of [HB(pz)_3_]Ag(PCN) (**4**) in different solvents at room temperature. Figure S9: ^31^P NMR spectra of [HB(pz)_3_]Ag(PPh_3_) (**5**) in CDCl_3_ and DMSO at room temperature. Figure S10: Full ESI(+)-MS spectra of: (a) [HB(pz)_3_]Ag(PTA), **1**; (b) [HB(pz)_3_]Ag(DAPTA), **2**; (c) [HB(pz)_3_]Ag(PTA-SO_2_), **3**. Figure S11: Full ESI(+)-MS spectra of: (a) [HB(pz)_3_]Ag(PCN), **4**; (b) [HB(pz)_3_]Ag(PPh_3_), **5**. Figure S12:^1^H NMR and ^31^P NMR spectra of [Ag(DAPTA)_4_]BF_4_ (**7**) in D_2_O. Figure S13: ^1^H NMR and ^31^P NMR spectra of [Ag(PTA-SO_2_)_4_]BF_4_ (8) in DMSO. Figure S14: Full ESI(+)-MS spectrum of [Ag(DAPTA)_4_]BF_4_ (**7**). Figure S15: Full ESI(+)-MS spectrum of [Ag(PTA-SO_2_)_4_]BF_4_ (8) Figure S16A: Electronic spectra of CT-DNA in Tris-HCl buffer upon addition of increasing concentrations of **5** (a) and **10** (b) silver(I) complexes. [Compound] = 0–60 μM, [DNA] = 30 μM. Figure S16B: Stern–Volmer quenching plots of tested compounds 1–10.
