# Supplementary material for: Antiproliferative Homoleptic and Heteroleptic Phosphino Silver(I) Complexes: Effect of Ligand Combination on Their Biological Mechanism of Action
Source: Molecules. 2020 Nov 23;25(22):5484. doi: 10.3390/molecules25225484 (PMC7700221; doi:10.3390/molecules25225484)
Supplement: Supplementary file 1 [file molecules-25-05484-s001.pdf]

Antiproliferative Homoleptic and Heteroleptic Phosphino Silver(I) Complexes: effect of Ligand Combination on their Biological Mechanism of Action

Khouloud Dammak [1], Marina Porchia[2*], Michele De Franco[3], Mirella Zancato[3], Houcine Naïli[1], Valentina Gandin[3*], Cristina Marzano[3]

[1]Laboratoire Physico-Chimie de l'Etat Solide, Département de Chimie, Faculté des Sciences de Sfax, B.P. 1171, 3000 Sfax, Université de Sfax, Tunisie
[2]CNR-ICMATE, Corso Stati Uniti 4, 35127 Padova, Italy
3Department of Pharmaceutical and Pharmacological Sciences, University of Padova, via Marzolo 5, 35131 Padova, Italy
*Correspondence: valentina.gandin@unipd.it; marina.porchia@cnr.it

EXPERIMENTAL SECTION

Characterization of silver complexes

^{1}H, ^{31}P and ^{13}C spectra were recorded on a Bruker AMX-300 instrument (300.13 MHz for ^{1}H, 121.41 MHz for ^{31}P, 75.47 MHz for ^{13}C), using $SiMe_4$ as internal reference (^{1}H and ^{13}C) and 85% aqueous H_3PO_4 as external reference (^{31}P). FT IR spectra were recorded on a Mattson 3030 Fourier transform spectrometer in the range 4000-400 cm^{-1} in KBr pellets. Mass spectra have been recorded by an electrospray LCQ ThermoFinnigan mass spectrometer.
Some representative IR, NMR and mass spectra are reported in figures S2-S15.

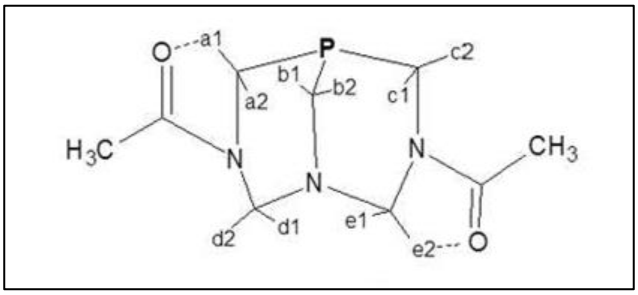

Figure S1. Numbering scheme of DAPTA ligand.

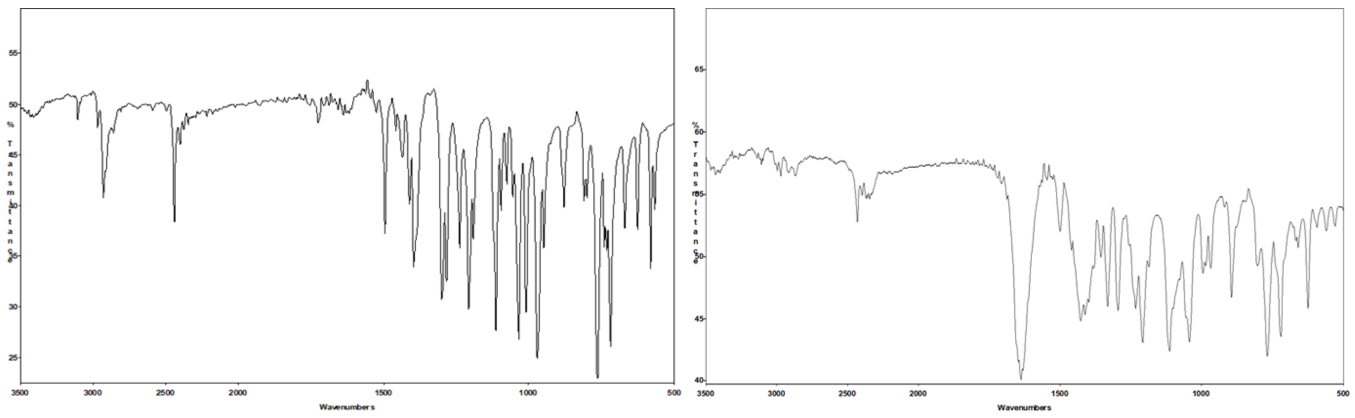

Figure S2. IR spectra in KBr of $[HB(pz)_3]Ag(PTA)$ **(1)** (left) and $[HB(pz)_3]Ag(DAPTA)$ **(2)** (right)

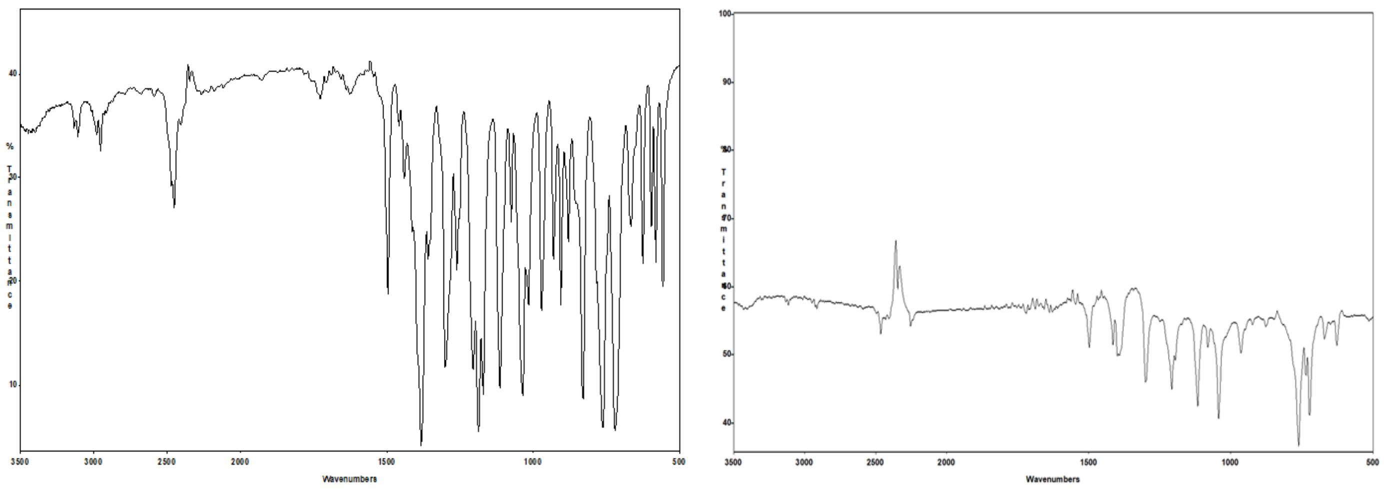

Figure S3. IR spectra in KBr of $[HB(pz)_3]Ag(PTA\text{-}SO_2)$ **(3)** (left) and $[HB(pz)_3]Ag(PCN)$ **(4)** (right)

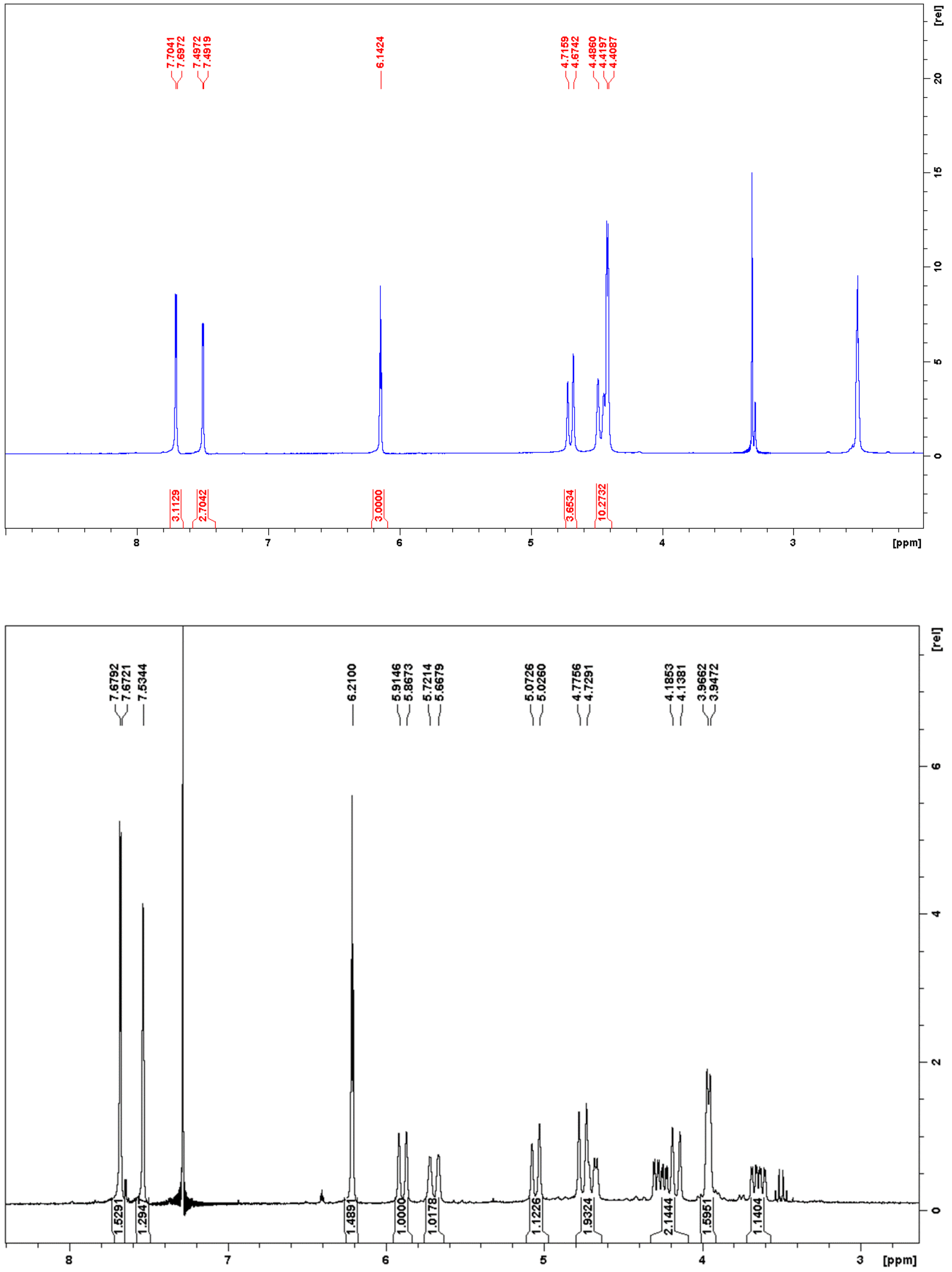

Figure S4. ^{1}H NMR spectra of [HB(pz)$_3$]Ag(PTA) **(1)** in DMSO (top) and of [HB(pz)$_3$]Ag(DAPTA) **(2)** in $CDCl_3$ (down).

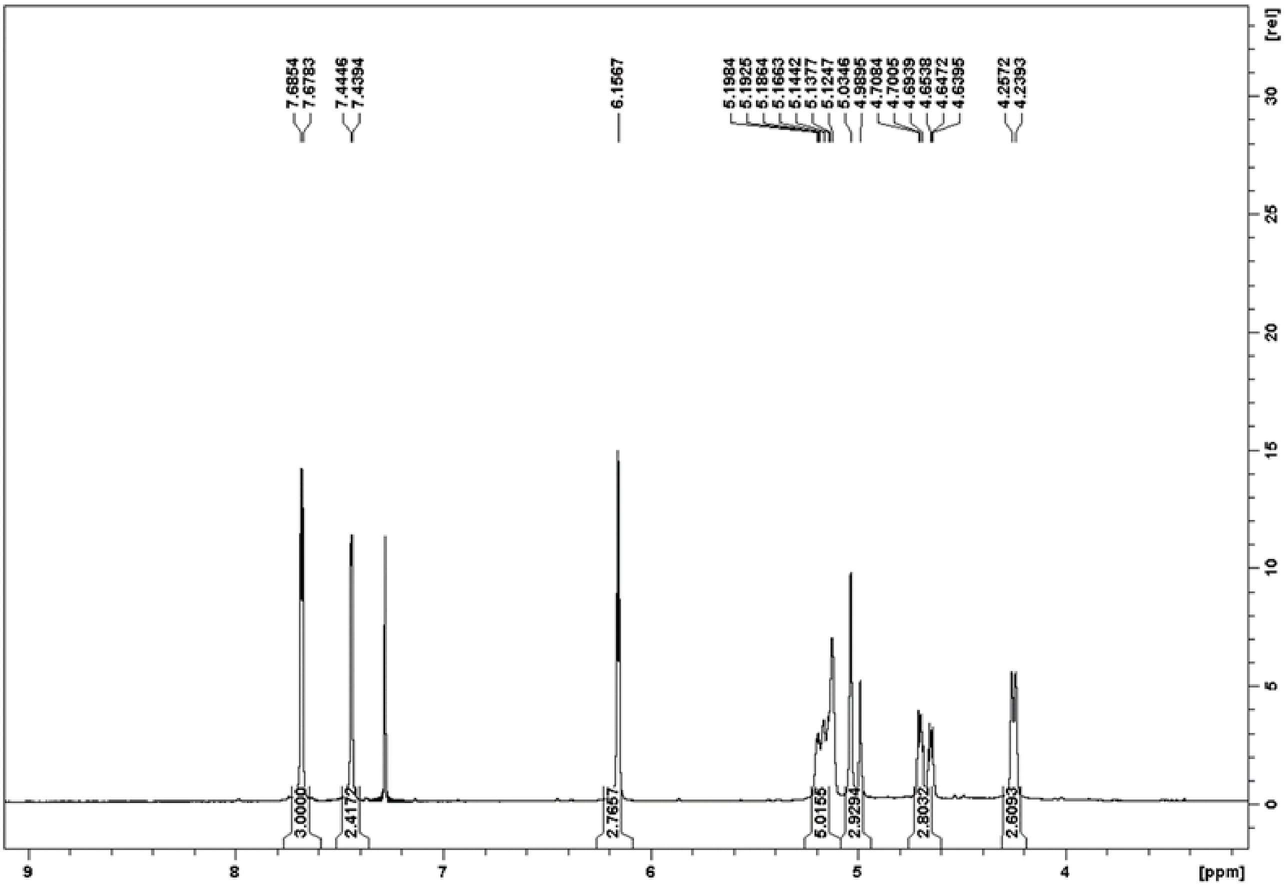

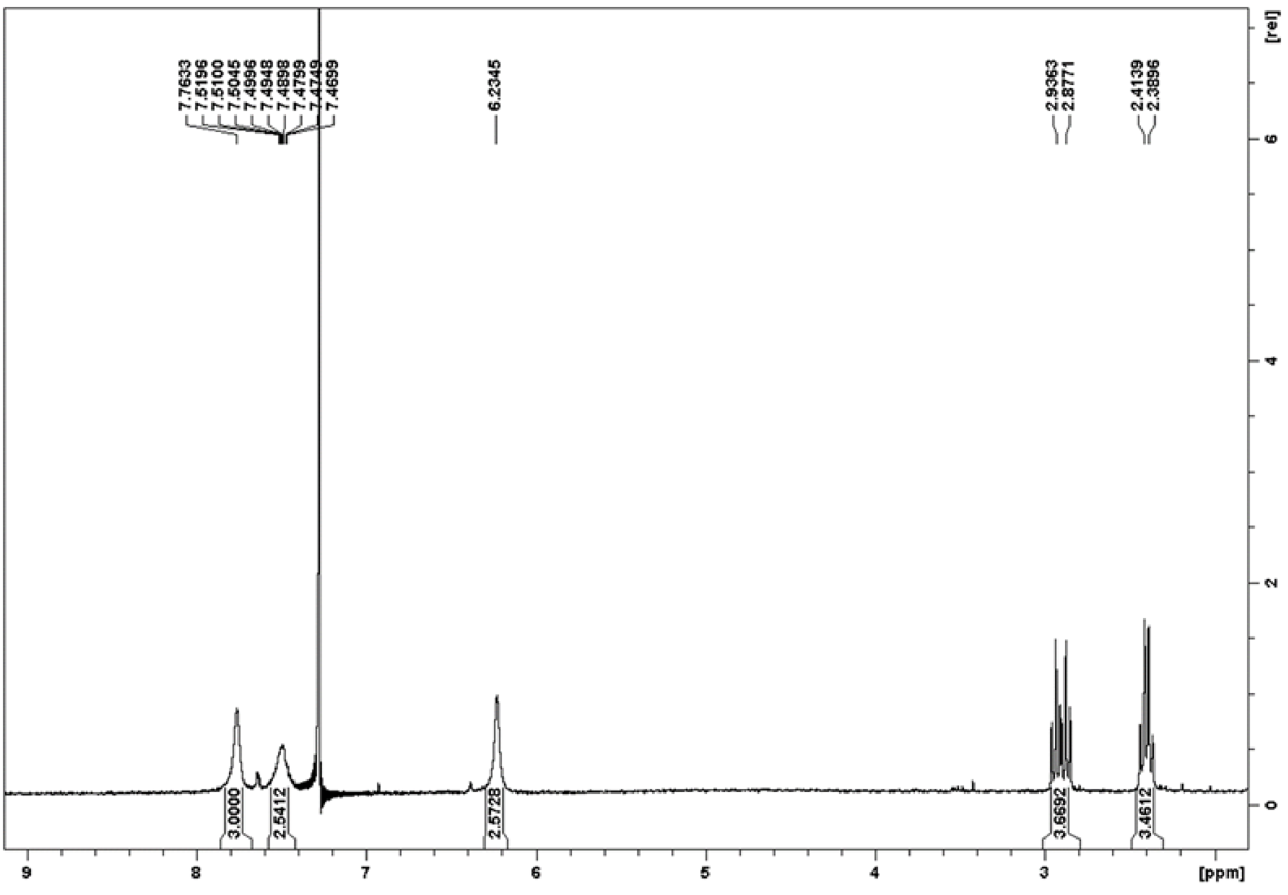

Figure S5. ^{1}H NMR spectra of $[HB(pz)_3]Ag(PTA\text{-}SO_2)$ **(3)** (top) and $[HB(pz)_3]Ag(PCN)$ **(4)** (down) in $CDCl_3$.

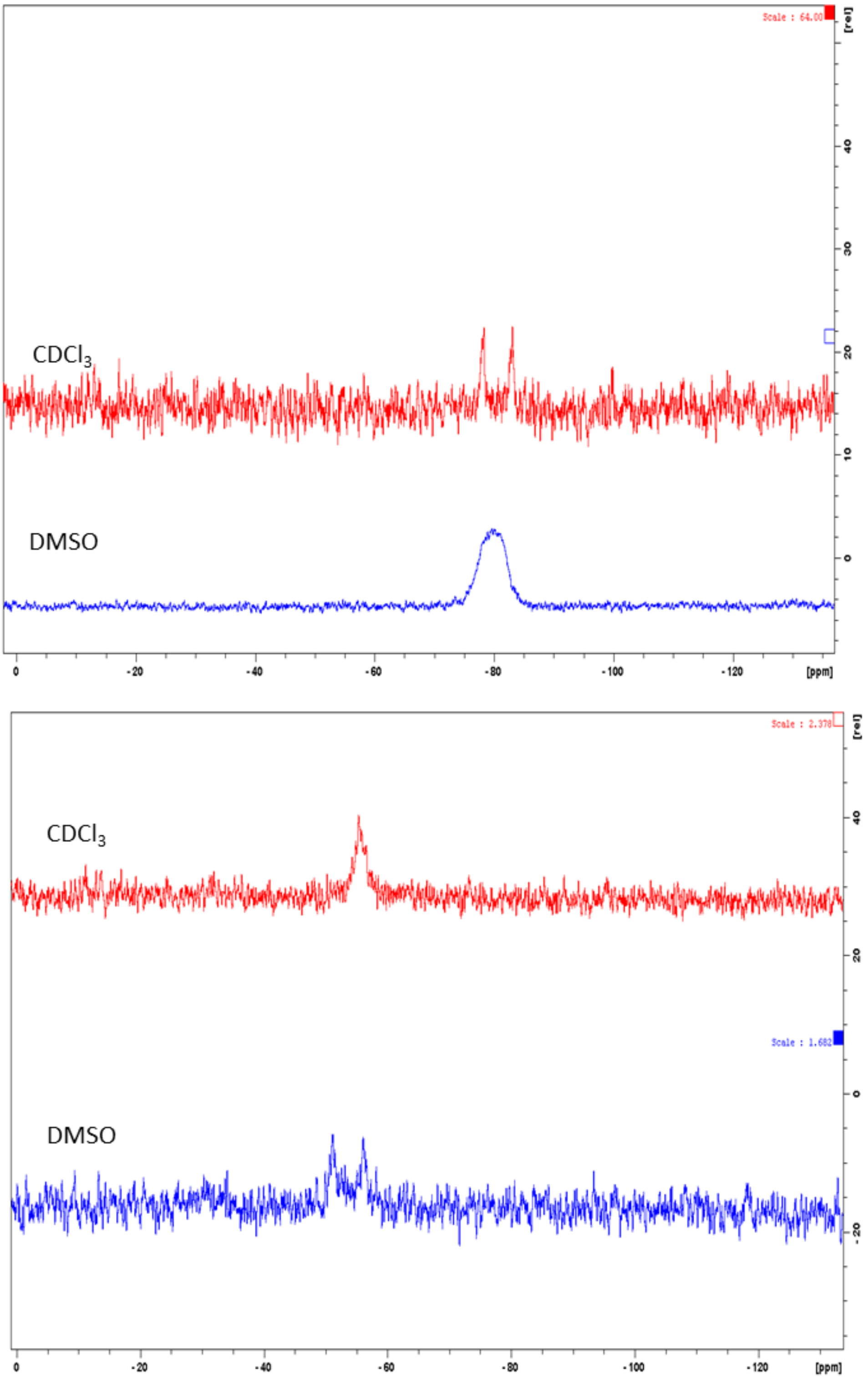

Fig. S6. ^{31}P NMR spectra of [HB(pz)$_3$]Ag(PTA) (**1**) (top) and [HB(pz)$_3$]Ag(DAPTA)(**2**) (down) in different solvents at room temperature.

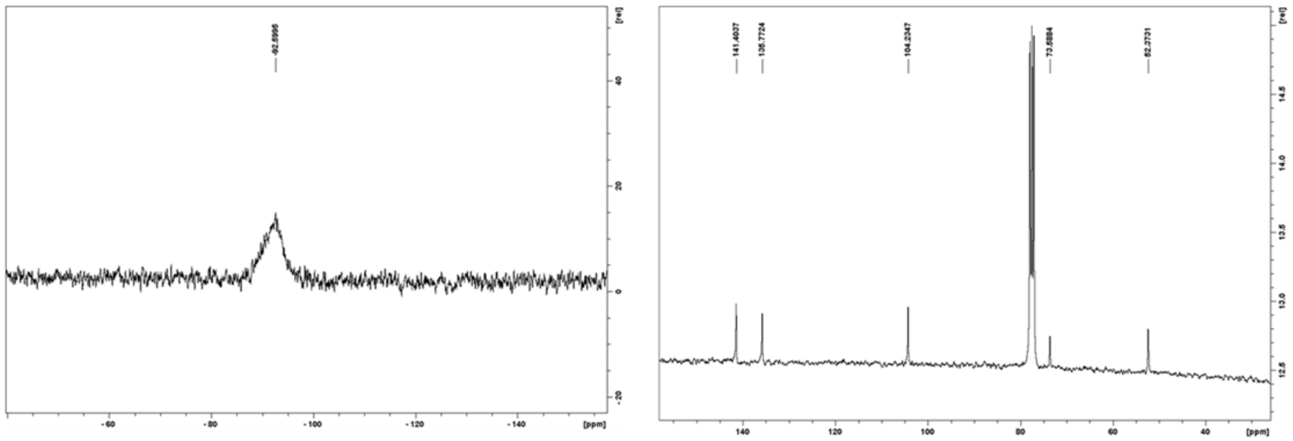

Figure S7. NMR characterization of $[HB(pz)_3]Ag(PTA\text{-}SO_2)$ **(3)** in $CDCl_3$: ^{31}P (left) and ^{13}C (right).

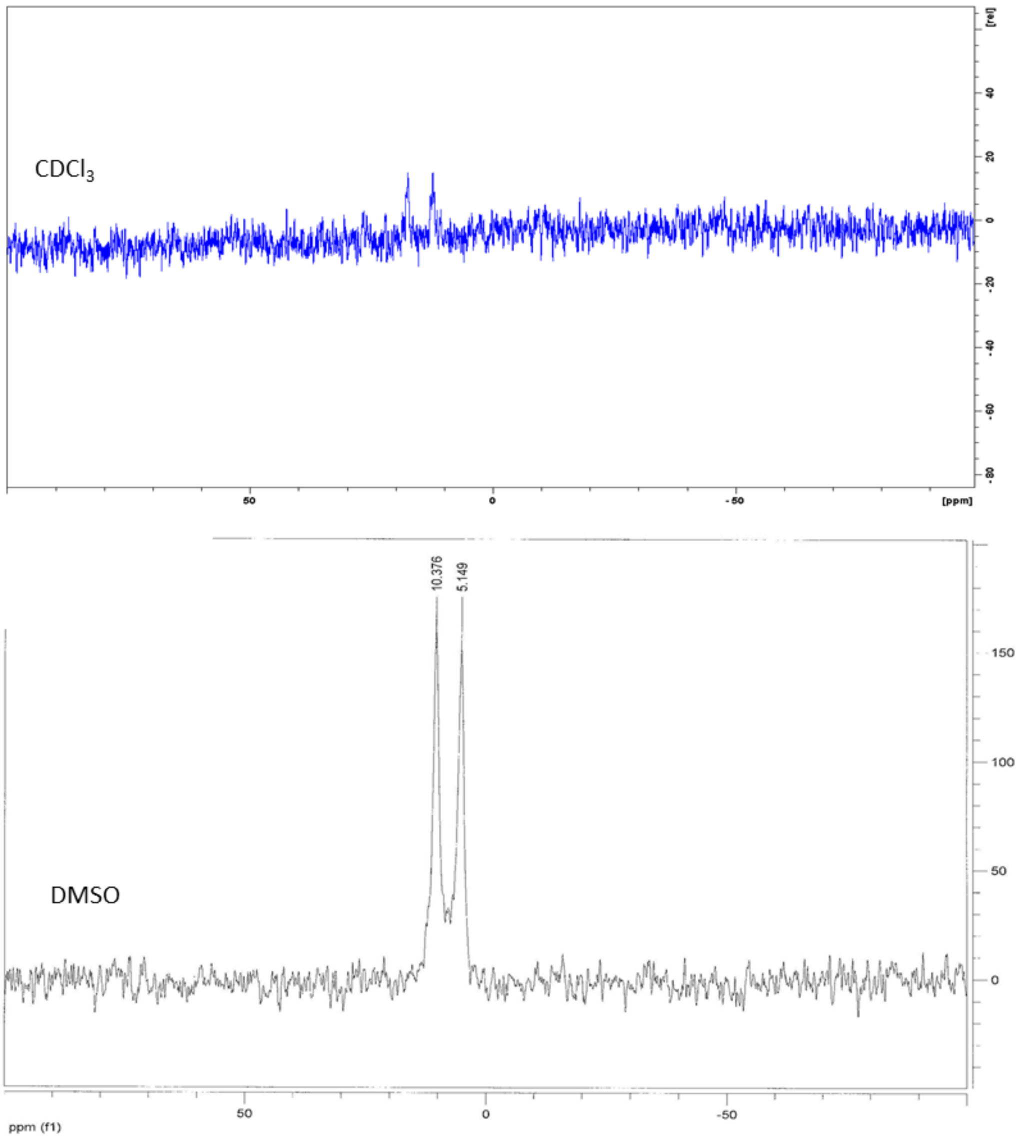

Figure S8. ^{31}P NMR spectra of [HB(pz)$_3$]Ag(PCN) **(4)** in different solvents at room temperature.

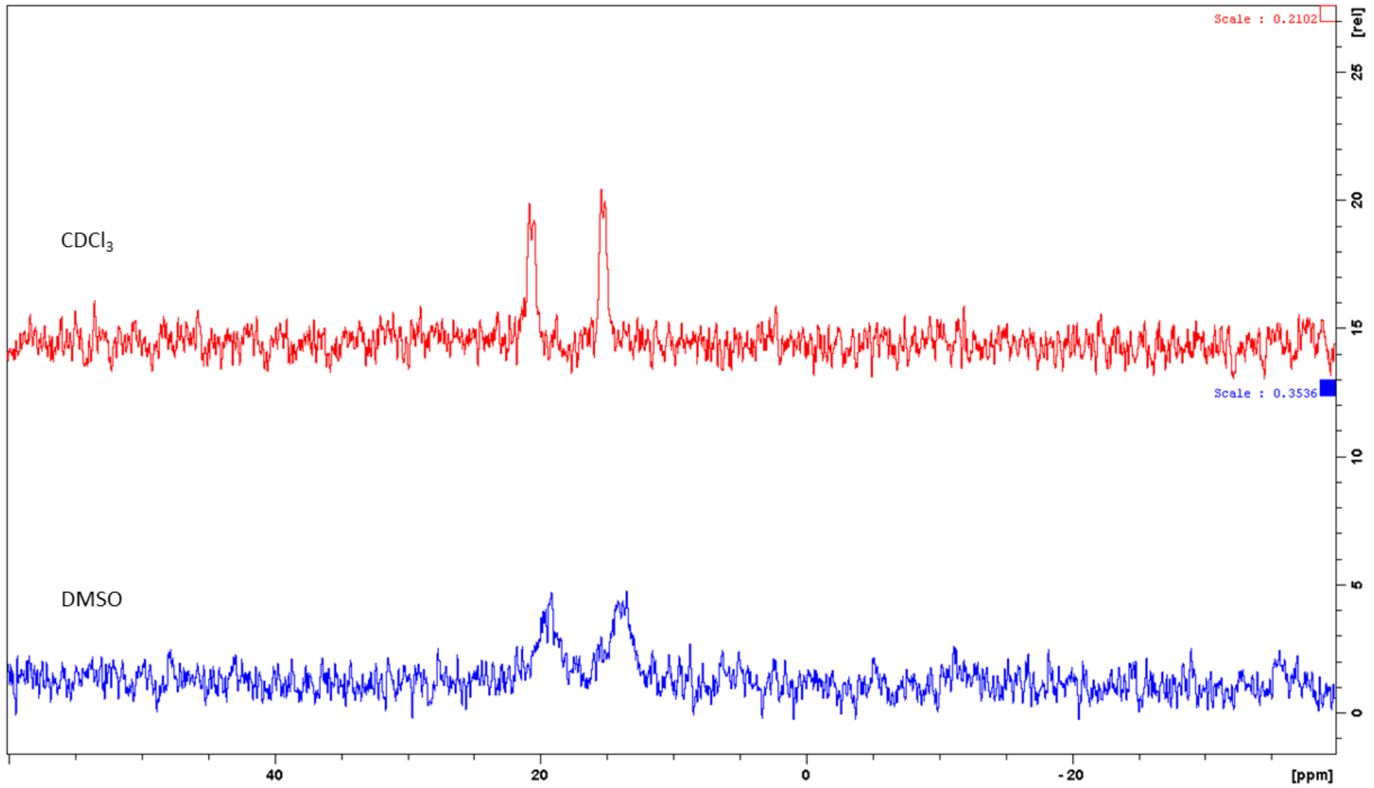

Figure S9. ^{31}P NMR spectra of [HB(pz)$_3$]Ag(PPh$_3$) (**5**) in $CDCl_3$ (top) and DMSO (down) at room temperature.

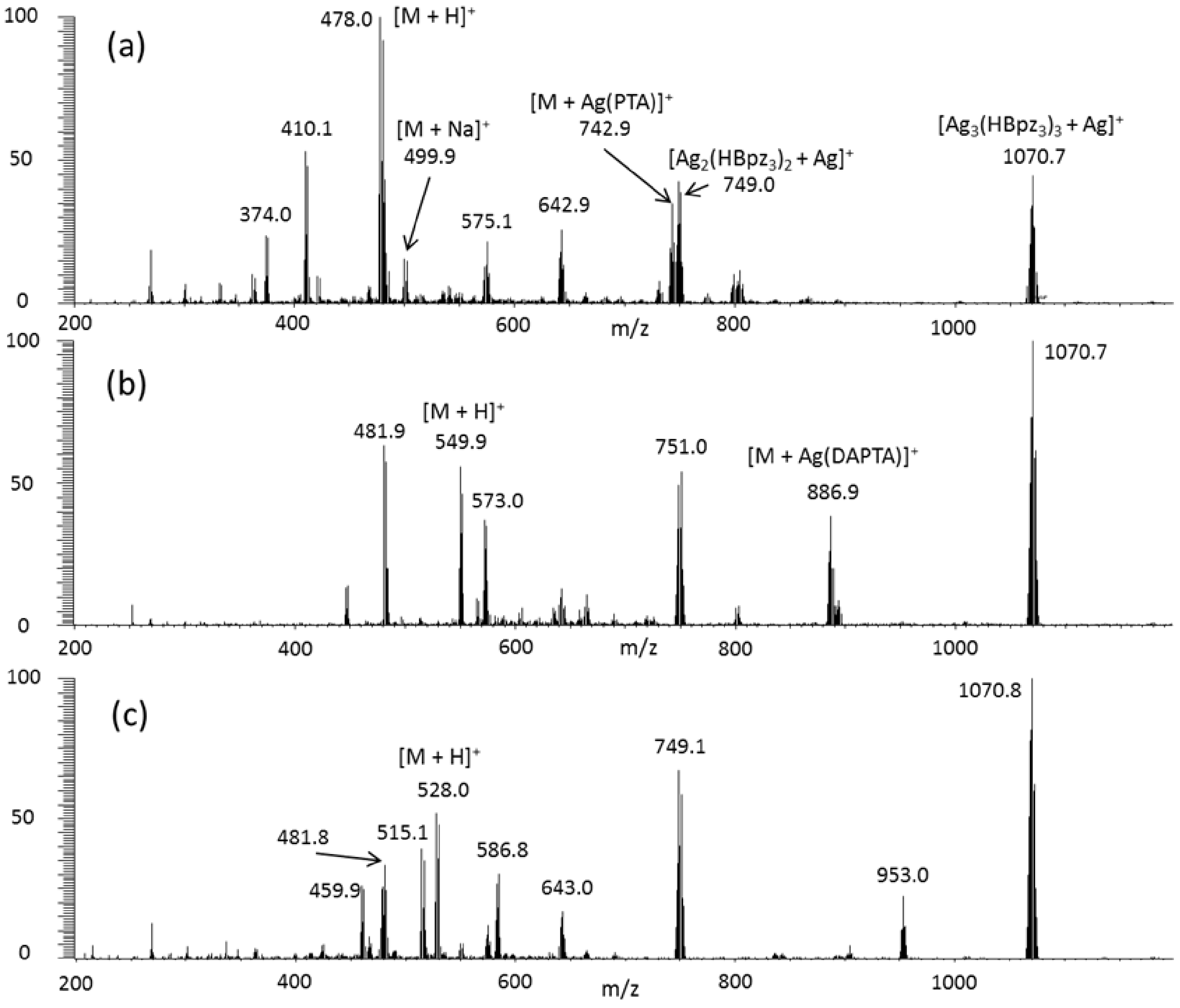

Figure S10. Full ESI(+)-MS spectra of: (a) $[HB(pz)_3]Ag(PTA)$, **1**; (b) $[HB(pz)_3]Ag(DAPTA)$, **2**; (c) $[HB(pz)_3]Ag(PTA\text{-}SO_2)$, **3**.

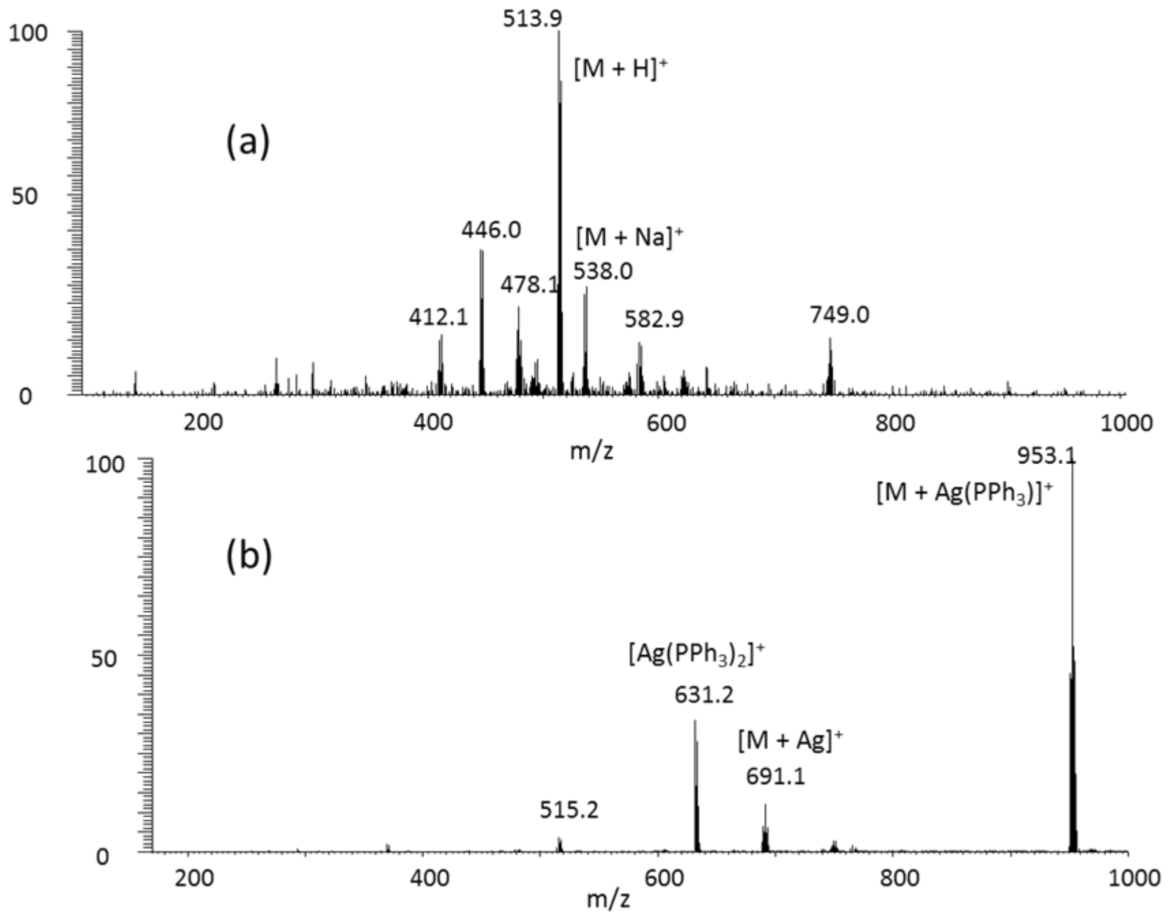

Figure S11. Full ESI(+)-MS spectra of: (a) $[HB(pz)_3]Ag(PCN)$, **4**; (b) $[HB(pz)_3]Ag(PPh_3)$, **5**.

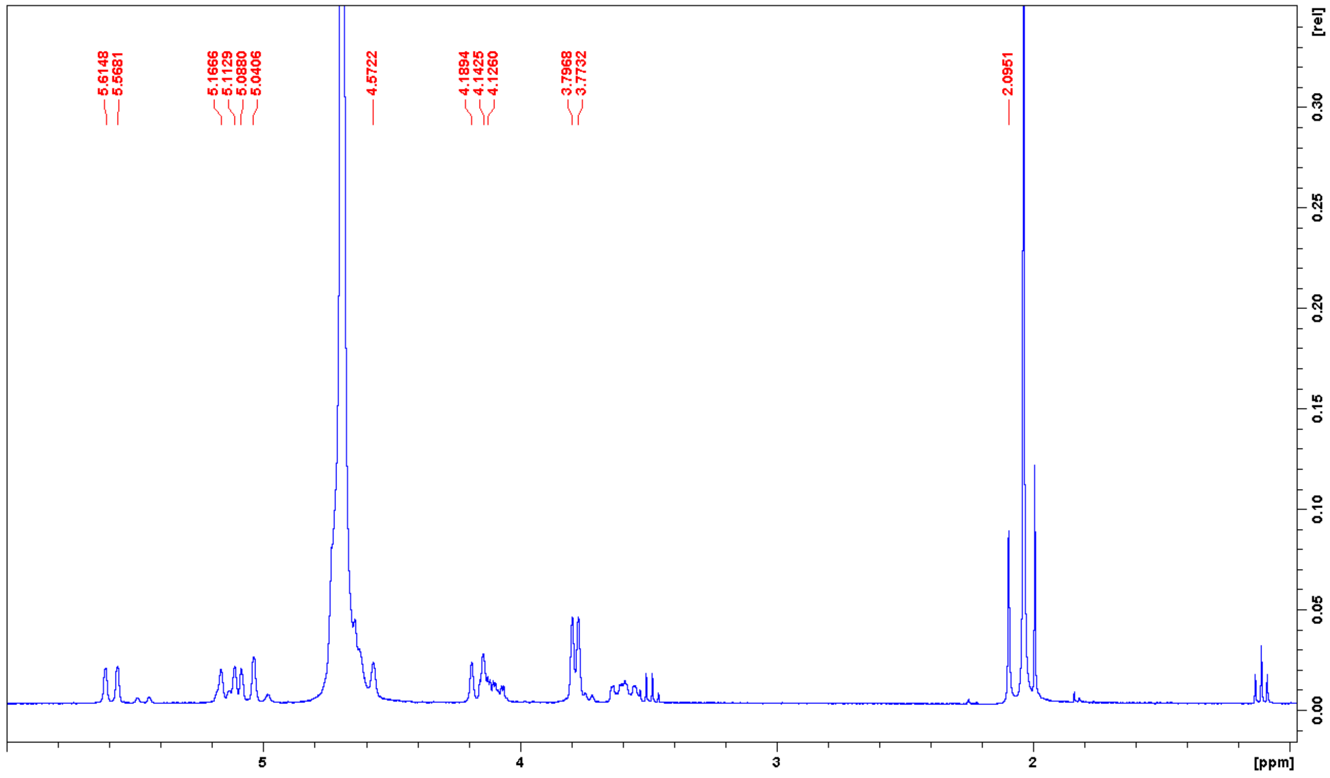

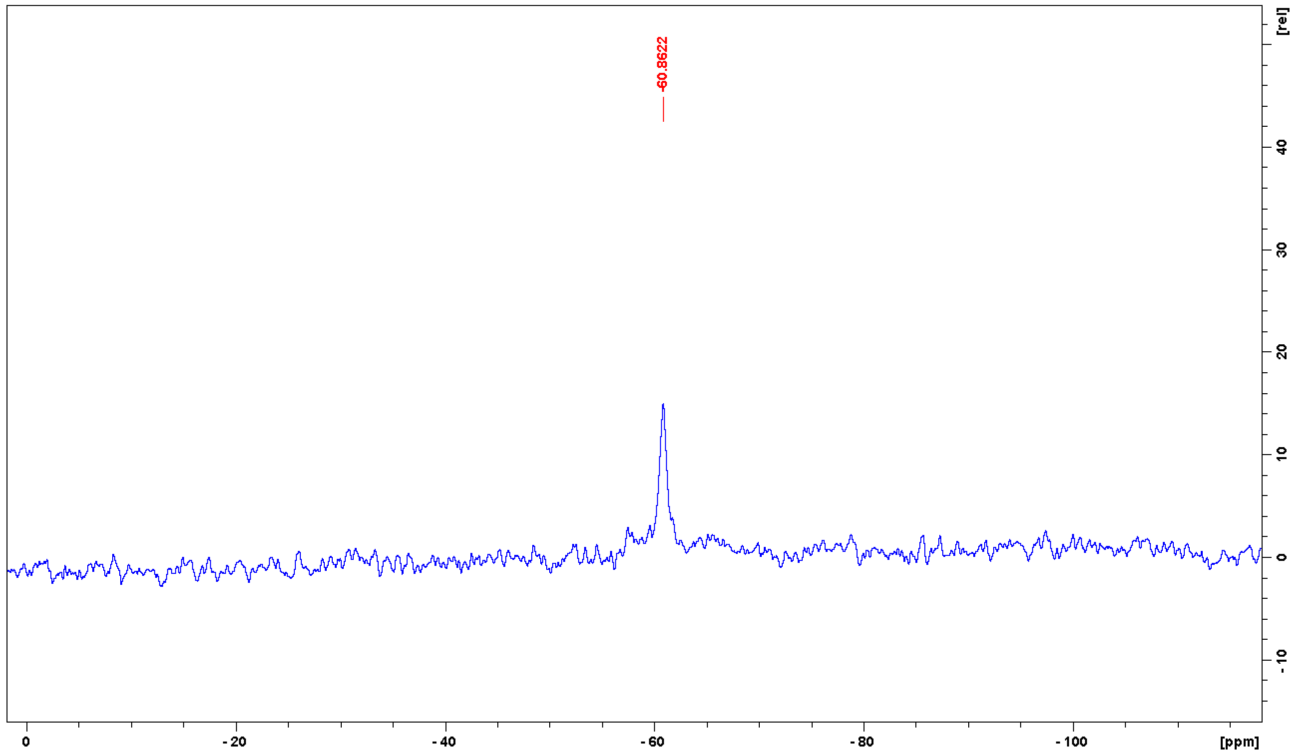

Figure S12. ^{1}H NMR spectra (top) and ^{31}P NMR spectra (down) of $[Ag(DAPTA)_4]BF_4$ **(7)** in D_2O.

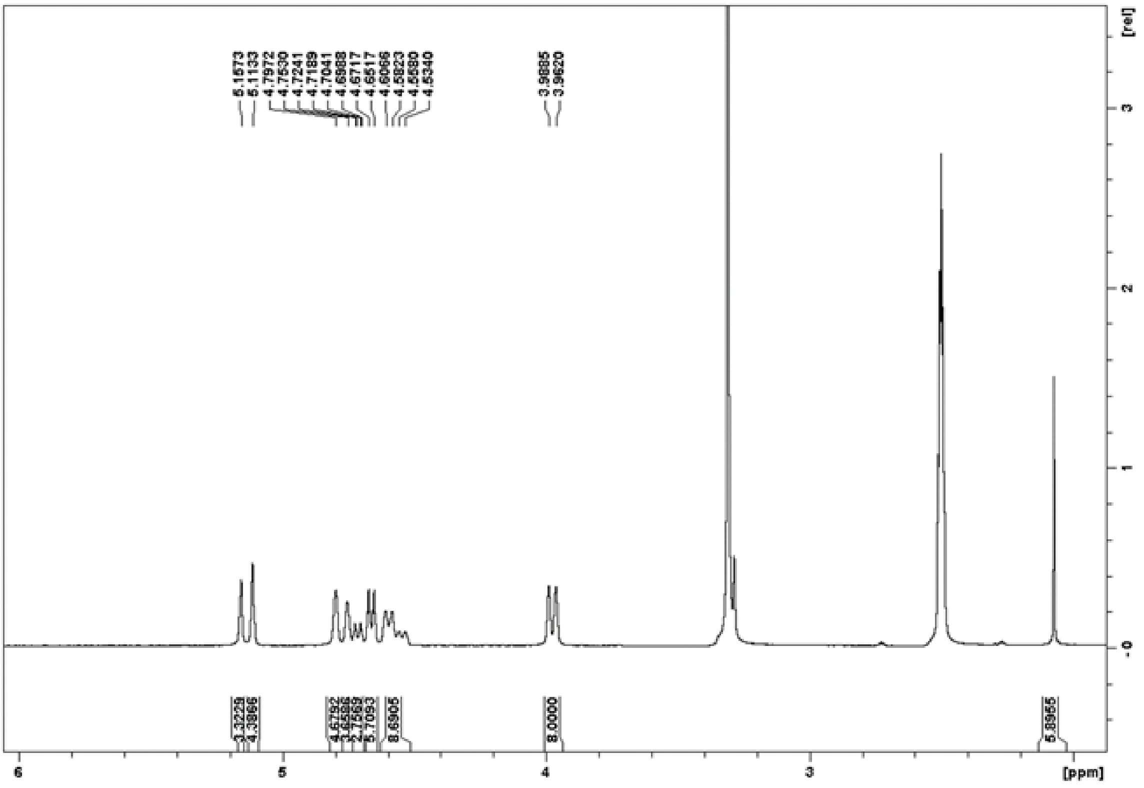

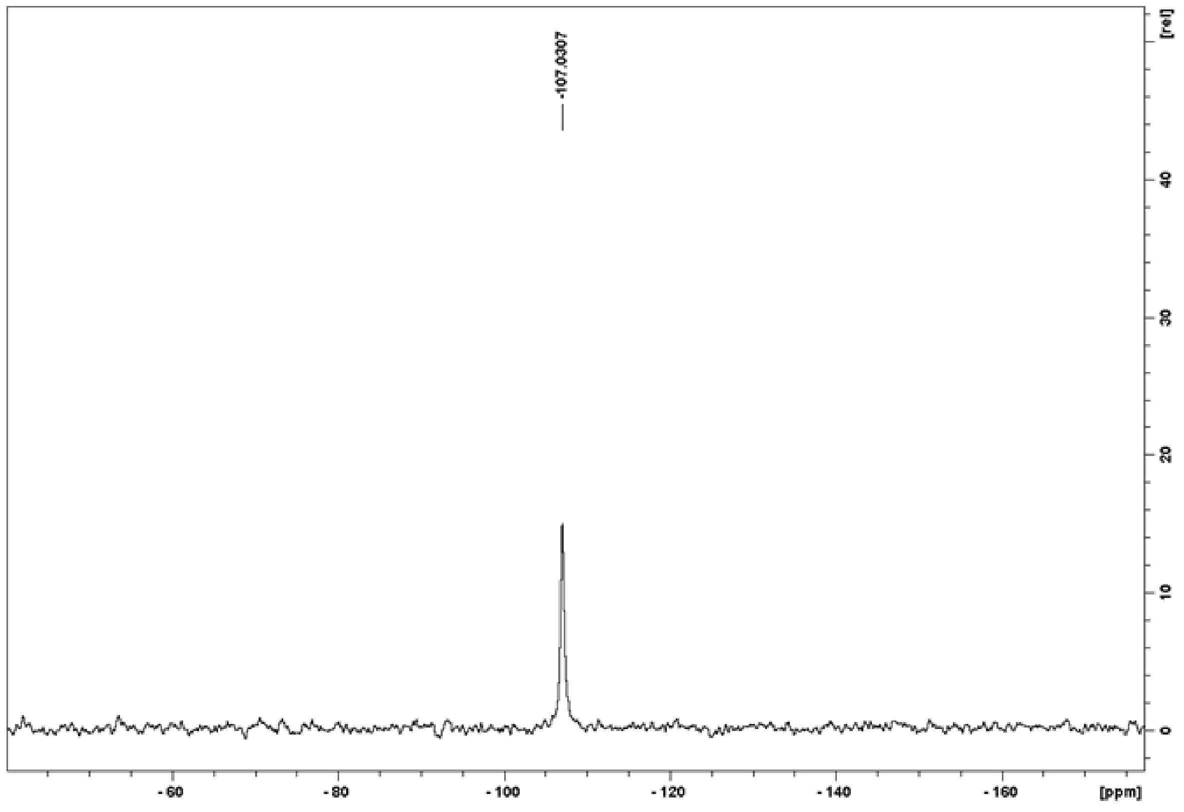

Figure S13. ^{1}H NMR spectra (top) and ^{31}P NMR spectra (down) of $[Ag(PTA\text{-}SO_2)_4]BF_4$ **(8)** in DMSO.

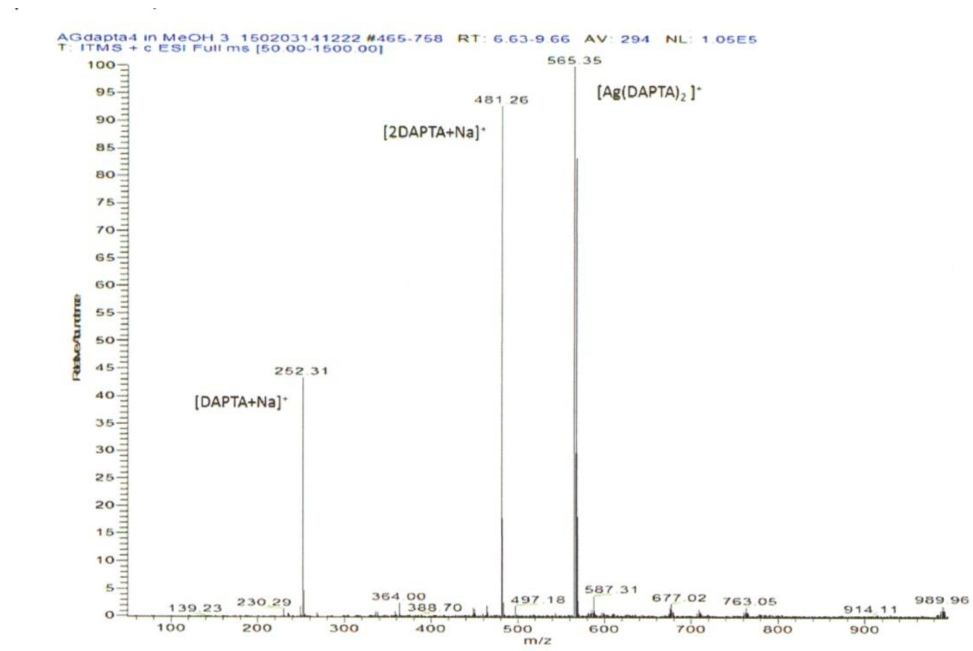

Figure S14. Full ESI(+)-MS spectrum of **[**$Ag(DAPTA)_4]BF_4$ **(7)**

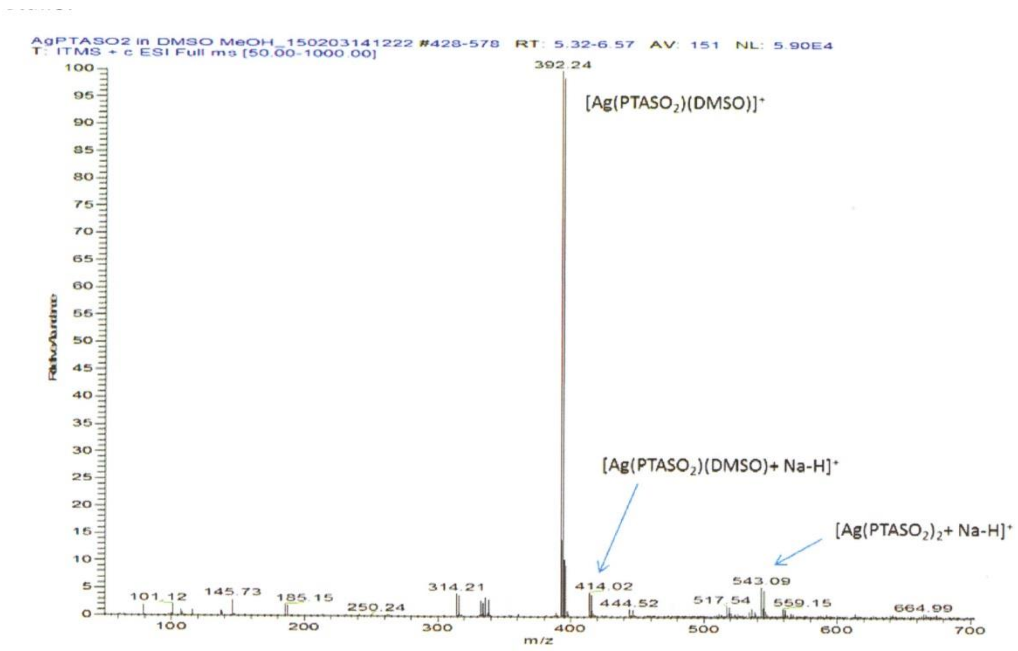

Figure S15. Full ESI(+)-MS spectrum of **[**$Ag(PTA\text{-}SO_2)_4]BF_4$ **(8)**

DNA binding studies

The UV–Vis absorption spectroscopic studies and the DNA binding experiments were performed at room temperature. Experiments were performed by maintaining a fixed CT-DNA concentration (30 µM) and rising silver complex concentration (15-60 µM). Sample solutions were scanned in the range 200−500 nm on a computer-controlled Varian Coulter DU 800 spectrophotometer.

EB–DNA competition experiments

The ethidium bromide (EB) displacement experiments were performed by titration with aliquots of tested compounds of a Tris buffer solution containing 10 µM DNA (8.0–15 kbp) and 0.33 µM EB (saturated binding levels). The fluorescence spectrum of the solution was obtained by exciting at 520 nm and measuring the emission spectra at 587 nm. The quenching efficiency for each compound was evaluated according to the classical Stern–Volmer equation: $F_0/F = 1+K_{SV}r$, where F_0 is the emission intensity in the absence of quencher, F is the emission intensity in the presence of quencher, K_{SV} is the Stern–Volmer constant, and r is the concentration ratio of the complex to DNA. K_{SV} constant was determined from the slope of the diagram F_0/F versus r.

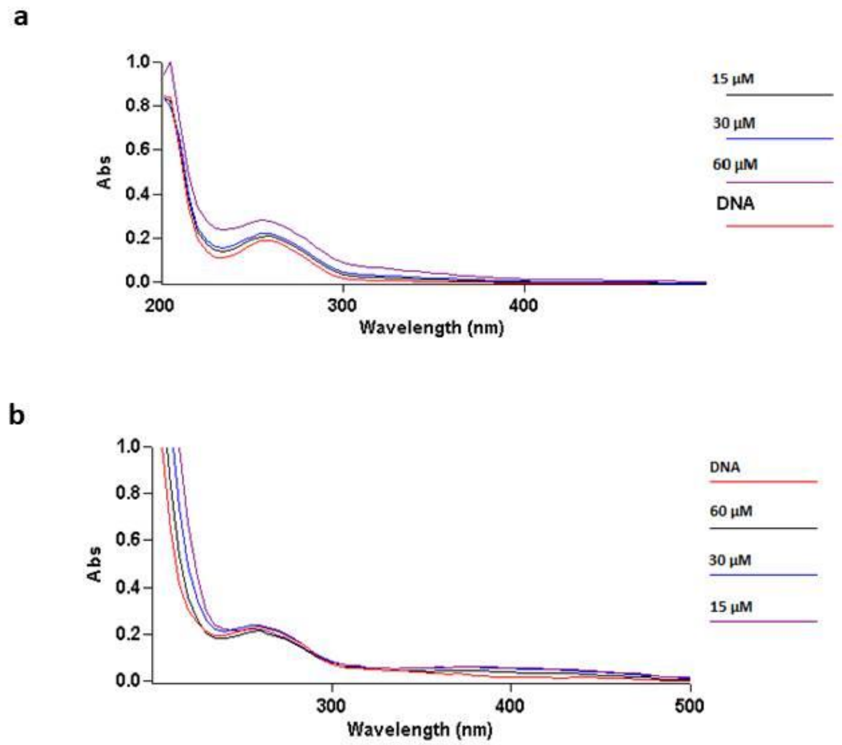

Figure S16A. Electronic spectra of CT-DNA in Tris-HCl buffer upon addition of increasing concentrations of **5** (a) and **10** (b) silver(I) complexes. [Compound]=0-60 μM, [DNA]=30 μM.

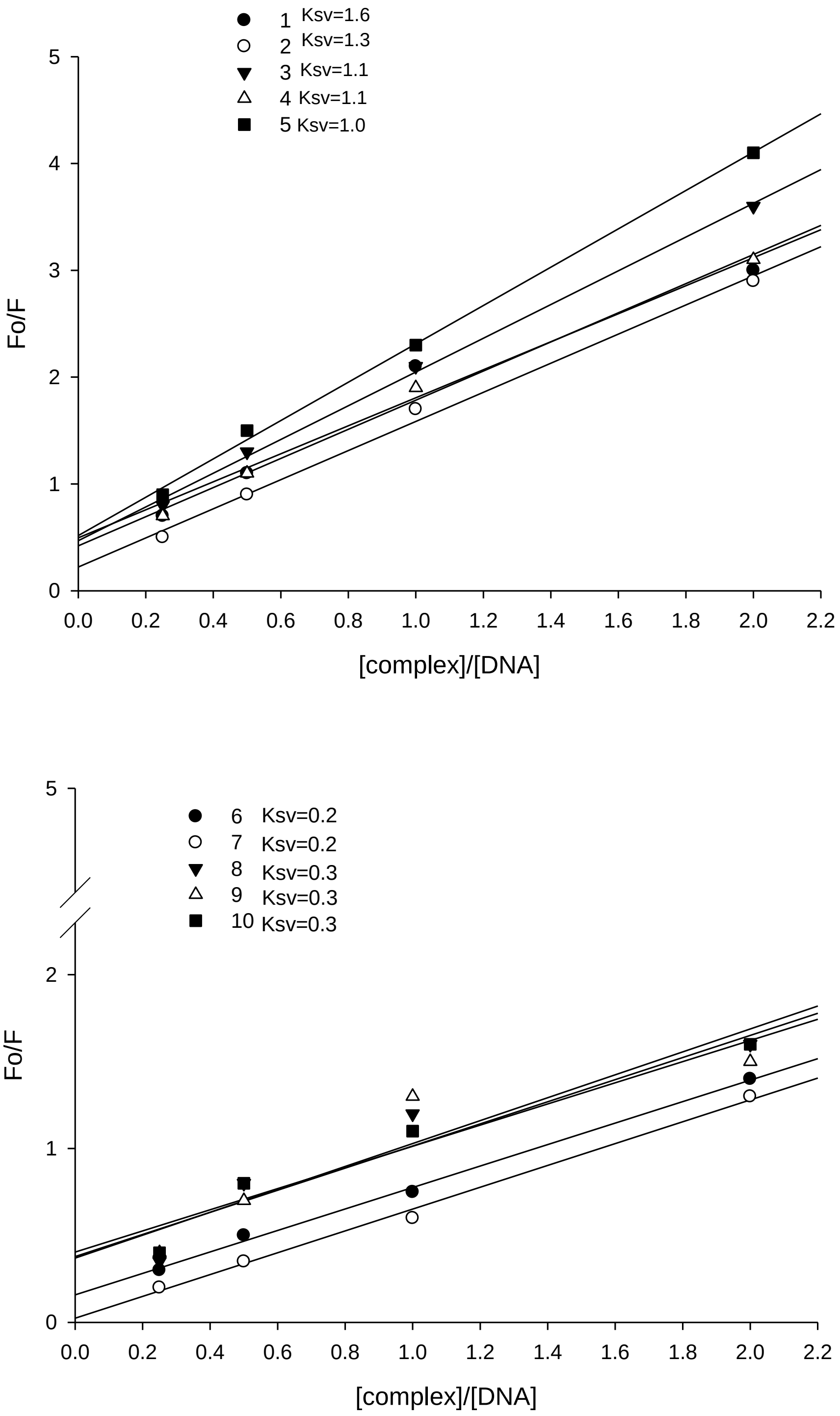

Figure S16B. Stern–Volmer quenching plots of tested compounds **1-10**.
